# Synthesis and antimicrobial activity of 1*H*-1,2,3-triazole and carboxylate analogues of metronidazole

**DOI:** 10.3762/bjoc.17.154

**Published:** 2021-09-09

**Authors:** Satya Kumar Avula, Syed Raza Shah, Khdija Al-Hosni, Muhammad U. Anwar, Rene Csuk, Biswanath Das, Ahmed Al-Harrasi

**Affiliations:** 1 Natural and Medical Sciences Research Center, University of Nizwa, P.O. Box 33, Postal Code 616, Birkat Al Mauz, Nizwa, Sultanate of Oman; 2 Organic Chemistry, Martin-Luther-University Halle-Wittenberg, Kurt-Mothes-Str. 2, d-06120, Halle (Saale), Germany

**Keywords:** antimicrobial agents, carboxylate analogues, 1*H*-1,2,3-triazole analogues, metronidazole, synthesis

## Abstract

Herein, a series of novel 1*H*-1,2,3-triazole and carboxylate derivatives of metronidazole (**5a–i** and **7a–e**) were synthesized and evaluated for their antimicrobial activity in vitro. All the newly synthesized compounds were characterized by ^1^H NMR, ^13^C NMR, HRMS, and ^19^F NMR (**5b**, **5c** and **5h**) spectroscopy wherever applicable. The structures of compounds **3**, **5c** and **7b** were unambiguously confirmed by single crystal X-ray analysis diffraction method. Single crystal X-ray structure analysis supported the formation of the metronidazole derivatives. The antimicrobial (antifungal and antibacterial) activity of the prepared compounds was studied. All compounds (except **2** and **3**) showed a potent inhibition rate of fungal growth as compared to control and metronidazole. The synthetic compounds also showed higher bacterial growth inhibiting effects compared to the activity of the parent compound. Amongst the tested compounds **5b**, **5c**, **5e**, **7b** and **7e** displayed excellent potent antimicrobial activity. The current study has demonstrated the usefulness of the 1*H*-1,2,3-triazole moiety in the metronidazole skeleton.

## Introduction

**Figure 1:**
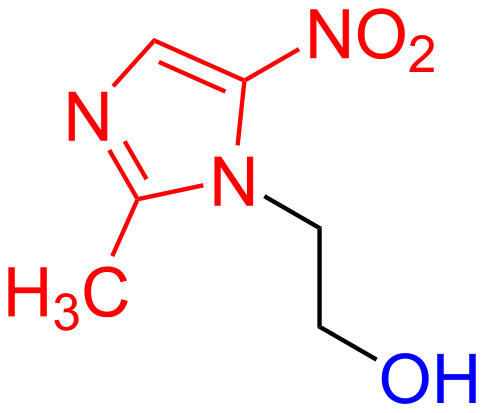
Structure of metronidazole (**1**).

Metronidazole (**1**) is an important antimicrobial agent which has been clinically used successfully for a long time. It was originally used for the treatment of infections caused by *Trichomonas varginalis* and later it was applied to treat various other infections [1]. For the last 45 years metronidazole (**1**) is in extensive use for the management of anaerobic infections. The compound possesses a broad spectrum of activity against various Gram-positive as well as Gram-negative organisms [2]. It is also a cost-effective drug. Due to its impressive antimicrobial activity and limited adverse effect metronidazole (**1**) has been considered as a “Gold Standard” antibiotic (Figure 1).

However, to avoid the problem related to clinical resistance to this antimicrobial agent some novel and improved analogues of this compounds are required. In this regard we suggested the modification of the alcohol tail of metronidazole by incorporating an *N*-heterocyclic moiety.

Nitrogen-containing heterocycles play a vital role in agrochemicals and pharmaceuticals [3]. Among these heterocyclic systems, the 1*H*-1,2,3-triazoles are very important in organic chemistry due to their broad spectrum of applications in biochemical, biomedicinal, pharmaceuticals, and materials sciences [4]. Their chemistry underwent a substantial growth over the past decades [5]. They are widely used in industrial applications such as photographic materials, dyes, agrochemicals, photostabilizers, and corrosion inhibitors (copper alloys) [6]. Incorporation of the 1*H*-1,2,3-triazole moiety is well known to impact on the physical, chemical and biological potential properties of organic molecules. Due to this reason, many efforts have been exerted to develop new synthetic methodologies toward the 1*H*-1,2,3-triazole group containing organic entities.

However, earlier methods of the synthesis of aliphatic and aromatic esters of metronidazole are associated with different drawbacks such as long conversion times, low yields and preparation of their respective acid chlorides by using thionyl chlorides and these acid chlorides were then made to react with the -OH functionality of metronidazole to get different esters [7]. Here we report a convenient method for the synthesis of aliphatic and aromatic esters of metronidazole.

**Figure 2:**
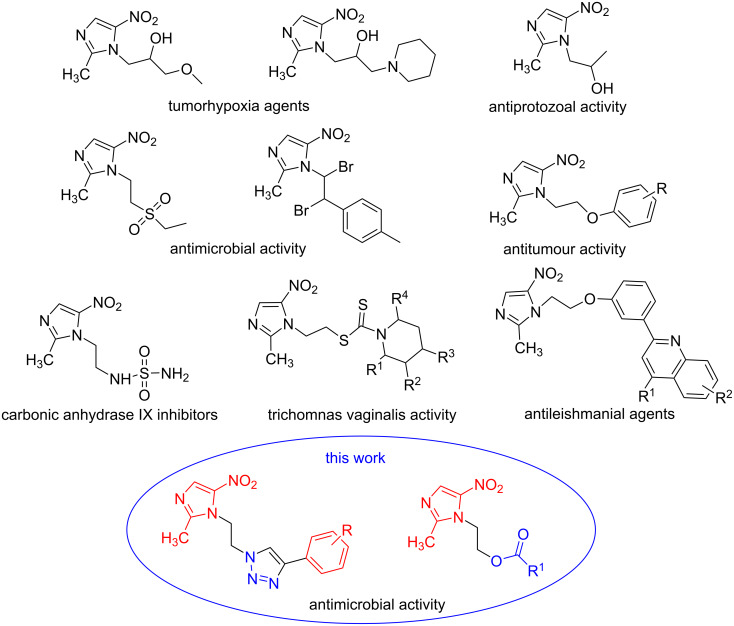
Chemical structures of some metronidazole derivatives with different biological activity.

Furthermore, derivatives of metronidazole scaffolds are known to have a large range of biological activities including tumorhypxia agents [8], antiprotozoal activity [9], antimicrobial [10], antitumour [11], carbonic anhydrase IX inhibitors [12], trichomonas vaginalis activity [13], antileishmanial agents [14] (Figure 2). We have recently synthesized several 1*H*-1,2,3-triazole-containing molecules with impressive biological activities [15].

In continuation of our research work on 1*H*-1,2,3-triazole derivatives [16], we have synthesized a series of new 1*H*-1,2,3-triazole and carboxylate derivatives of metronidazole (**5a–i** and **7a–e**). The choice of 1*H*-1,2,3-triazole was based on its known activities and its broad range of applications in biochemical, pharmaceutical, biomedicinal and materials sciences [4–5].

## Results and Discussion

### Chemistry: synthesis of 1*H*-1,2,3-triazole analogues of metronidazole

Metronidazole (**1**) has a free primary hydroxy group. The first step was initiated by the protection of the primary hydroxy group of metronidazole (**1**) with *p*-toluenesulfonyl chloride in dry DCM in the presence of triethylamine at 0 °C to room temperature. The reaction afforded the desired metronidazole tosylate **2** in high yield (96%) [17]. In the next step, the metronidazole tosylate **2** under treatment with NaN_3_ in DMF at 70 °C afforded the corresponding metronidazide **3** in 88% yield [18].

The ^1^H NMR spectrum of metronidazide **3** showed a singlet at δ 7.93 for the 1*H*-imidazole proton. Two triplet signals at δ 4.40 and δ 3.74 were assigned to four methylene protons of –N–CH_2_-CH_2_–N_3_. A singlet peak at δ 2.50 was due to methyl protons on the imidazole ring. The high-resolution mass spectrometric data at 197.0737 (M + H)^+^ confirmed the structure of metronidazide **3**.

**Figure 3:**
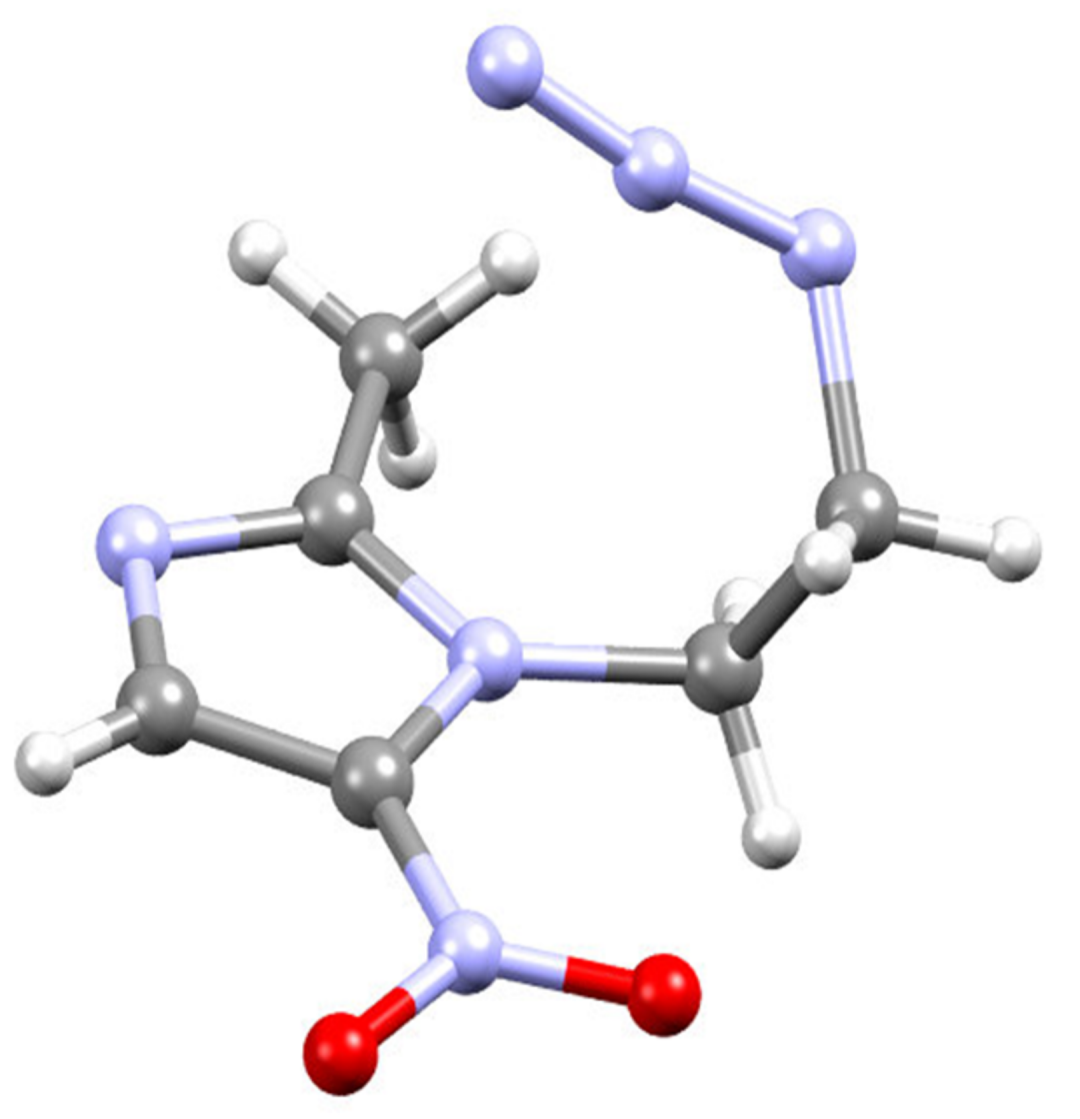
Crystal structure of compound **3**. Colour codes: carbon = grey, mitrogen = blue, oxygen = red, hydrogen = white.

Single crystals of metroazide compound **3** were grown from slow evaporation of DCM solution. The structure of metronidazide **3** was unambiguously confirmed by single crystal X-ray analysis (Figure 3).

The next step was carried out by using “click” chemistry involving the 1,3-dipolar cycloaddition reaction between metronidazide **3** and alkyne derivative **4a** in the presence of CuI and Hünig’s base with MeCN as a solvent. The reaction furnished the desired product metronidazole 1*H*-1,2,3-triazole derivative **5a** as a pale yellow solid in 85% yield [19–20].

**Scheme 1:**
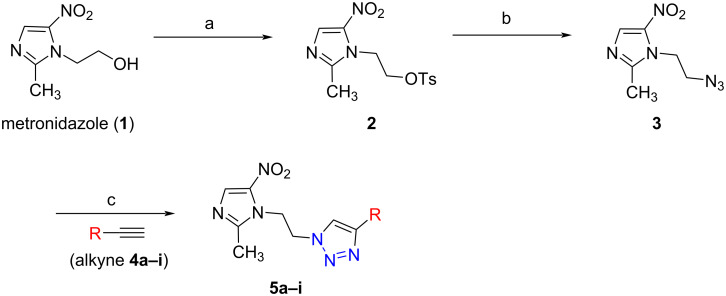
Reagents and conditions: (a) TsCl, Et_3_N, dry DCM, DMAP, 0 °C to room temperature, 5 h, 96%; (b) NaN_3_, DMF, 70 °C, 3 h, 88%; (c) alkyne derivative (**4a–i**), CuI, Et_3_N, CH_3_CN, room temperature, 3 h, (**5a–i**) 85–94%.

Similarly, using the same reaction conditions and procedure described for the synthesis of the 1*H*-1,2,3-triazole derivative of metronidazole **5a**, analogues **5b–i** were obtained in 86–94% yield using the different alkyne derivatives **4b–i**. The synthesis of the new 1*H*-1,2,3-triazole derivatives of metronidazole is summarized in Scheme 1 and Table 1.

**Table 1:** Synthesis of 1*H*-1,2,3-triazole compounds **5a**–**i**.

Alkyne reagents (**4**)	Compounds (**5**)	R	Yields of 1*H*-1,2,3-triazole products (**5**) (%)^a^

**a**	**a**	C_6_H_5_	85
**b**	**b**	4-CF_3_C_6_H_4_	90
**c**	**c**	4-FC_6_H_4_	92
**d**	**d**	COOMe	86
**e**	**e**	4-BrC_6_H_4_	89
**f**	**f**	4-NH_2_C_6_H_4_	87
**g**	**g**	4-CH_3_C_6_H_4_	90
**h**	**h**	2,4-F_2_C_6_H_3_	94
**i**	**i**	4-OMeC_6_H_4_	89

^a^Yields of isolated products.

Their chemical structures (**5a–i**) were confirmed by spectroscopic techniques (^1^H NMR, ^13^C NMR) and HRMS.

The ^1^H NMR spectrum of 1*H*-1,2,3-triazole compound **5c** showed two singlet signals at δ 8.13 and 7.99 corresponding to the 1*H*-imidazole and 1*H*-1,2,3-triazole protons, respectively. The four aromatic protons appeared in the region of δ 7.67–7.05 ppm. A doublet signal at δ 4.77 is due to the four methylene protons of –N–CH_2_-CH_2_–Ph. A singlet peak at δ 1.86 is attributed to methyl protons on the imidazole ring. The ^19^F NMR spectrum of 1*H*-1,2,3-triazole compound **5c** showed a singlet at δ −113.61 corresponding to one fluorine atom of the phenyl ring. The high-resolution mass spectrometric data at 317.1141 (M + H)^+^ supported the structure of 1*H*-1,2,3-triazole compound **5c**.

**Figure 4:**
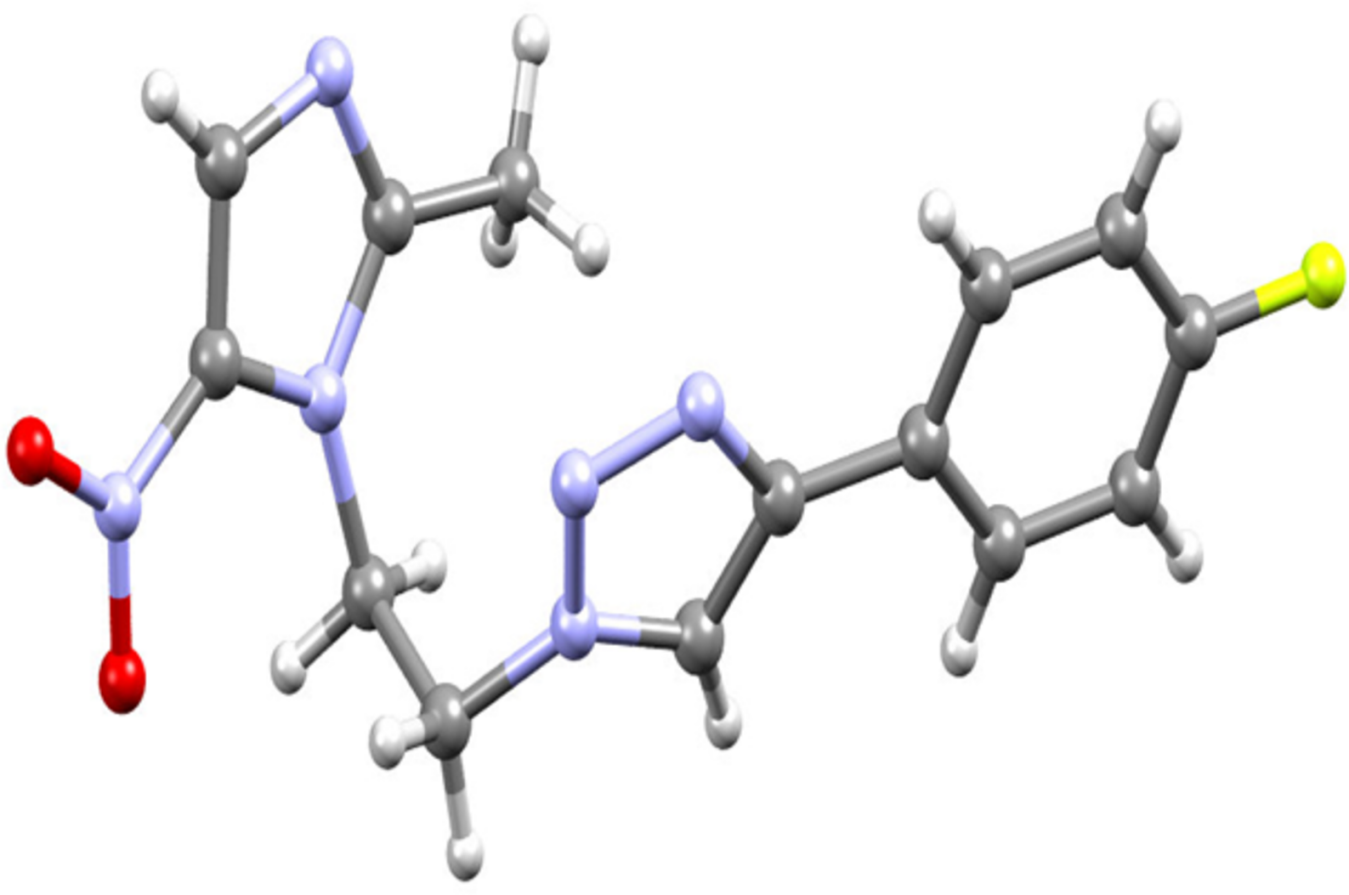
Crystal structure of 1*H*-1,2,3-triazole compound **5c**: Colour codes: carbon = grey, nitrogen = blue, oxygen = red, fluorine = yellow, hydrogen = white.

Single crystals of 1*H*-1,2,3-triazole compound **5c** were grown from slow evaporation of MeOH. The structure of 1*H*-1,2,3-triazole compound **5c** was unambiguously confirmed by single crystal X-ray analysis (Figure 4).

### Synthesis of carboxylate analogues of metronidazole

**Scheme 2:**
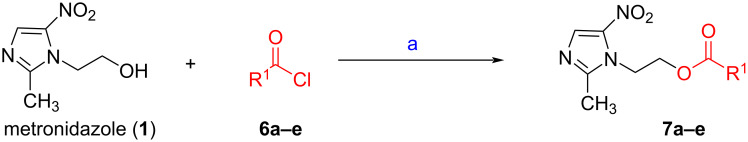
Reagents and conditions: (a) acid chlorides **6a–e**, pyridine, dry DCM, DMAP, room temperature, 4–5 h, 86–93%.

Compound **1** reacted with different acid chlorides (**6a–e**) in the presence of pyridine, a catalytic amount of DMAP and in dry DCM at room temperature. The reaction proceeded smoothly to give the desired metronidazole carboxylate derivatives **7a–e** in 86–93% yields [21–22]. The synthesis of the new metronidazole carboxylate derivatives is summarized in Scheme 2 and Table 2.

**Table 2:** Synthesis of carboxylate compounds **7a**–**e**.

Reagents (**6**)	Compounds (**7**)	R	Yield of **7** (%)^a^

**a**	**a**	C_6_H_5_	86
**b**	**b**	4-NO_2_C_6_H_4_	91
**c**	**c**	3,5-(NO_2_)_2_C_6_H_3_	93
**d**	**d**	C_2_H_5_	87
**e**	**e**	C_3_H_7_	89

^a^Yields of isolated compounds.

Their chemical structures (**7a–e**) were confirmed by spectroscopic techniques (^1^H NMR, ^13^C NMR and HRMS).

The ^1^H NMR spectrum of compound **7b** showed two doublet signals at δ 8.26 and 8.07 which are due to the four aromatic protons of the phenyl ring. A singlet signal at δ 7.95 is for the 1*H*-imidazole proton. Two doublet signals at δ 4.73 and δ 4.71 are assigned to the four methylene protons of –N–CH_2_-CH_2_–Ph. A singlet peak at δ 2.48 is due to the methyl protons on the imidazole ring. The high-resolution mass spectrometric data at 321.0842 (M + H)^+^ supported the structure of compound **7b**.

**Figure 5:**
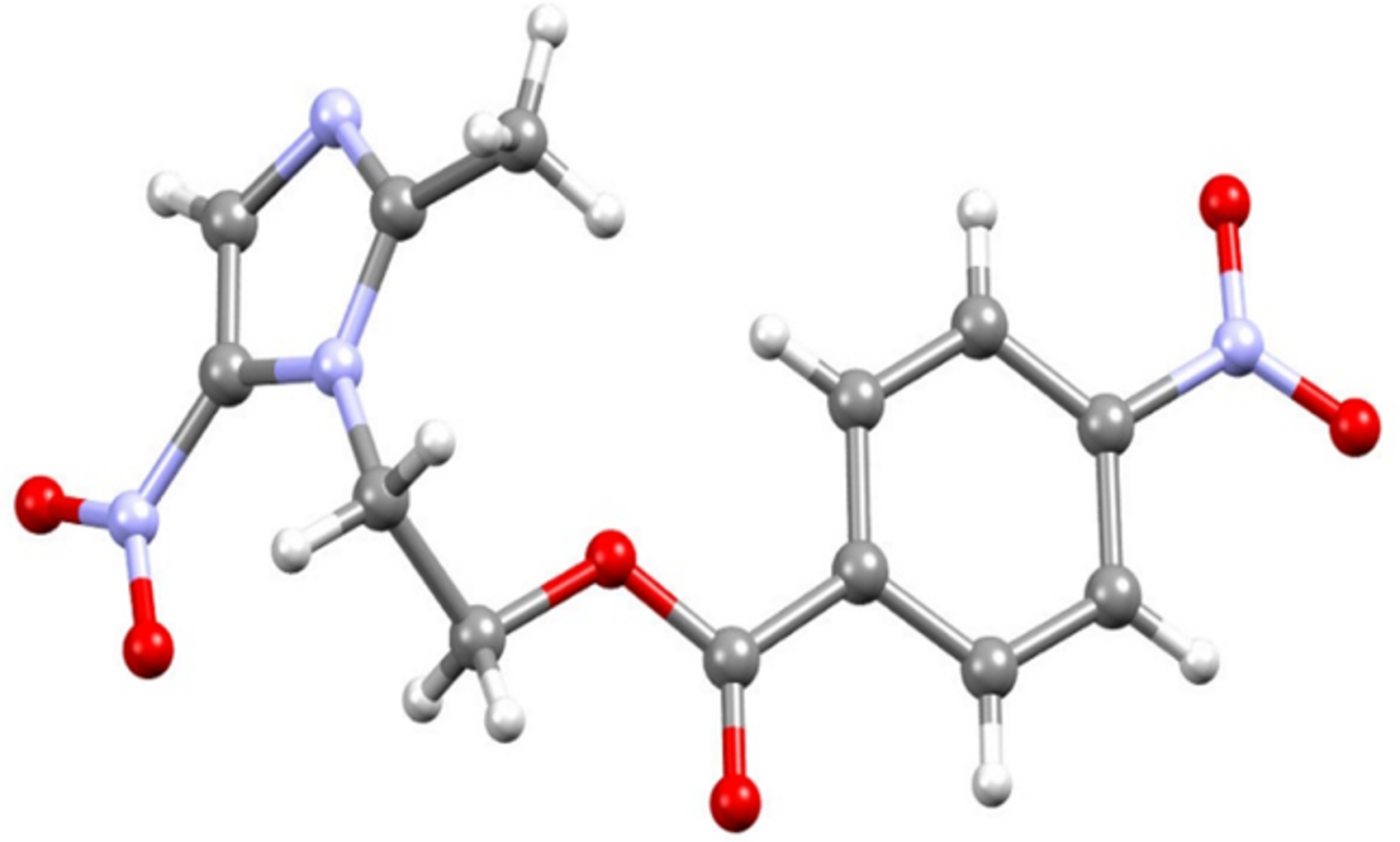
Crystal structures of compound **7b.** Colour codes: carbon = grey, nitrogen = blue, oxygen = red, hydrogen = white.

Single crystals of compound **7b** were grown from slow evaporation of MeOH + DCM (1:1) solution. The structure of compound **7b** was unambiguously confirmed by single crystal X-ray analysis (Figure 5).

In this article, chemical transformations of novel metronidazole 1*H*-1,2,3-triazole derivatives via “click” chemistry and carboxylate derivatives can lead to a wide range of biological applications.

### Antimicrobial activity

**Figure 6:**
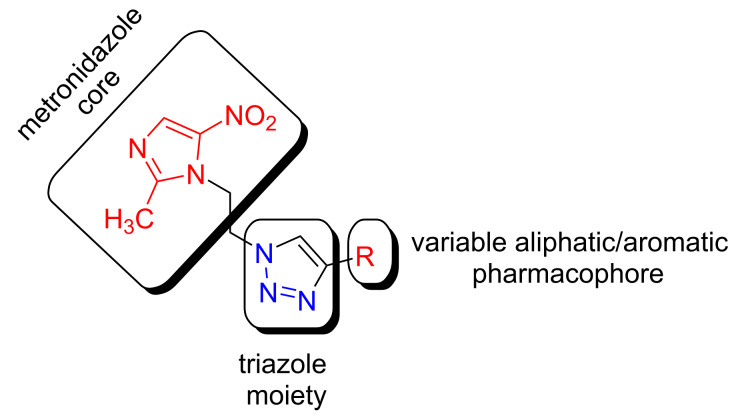
General structural feature of the synthesized molecules **5**.

The general structural pattern of the synthesized metronidazole derivatives is shown in Figure 6. Two pharmacophoric elements (metronidazole core and triazole moiety) were considered as rigid motif with an alkyl/aryl group attached to the triazole unit. A diverse array of functional groups in the aromatic ring influencing the antimicrobial activity of the molecules have been utilized.

### Antifungal activity of compounds

**Figure 7:**
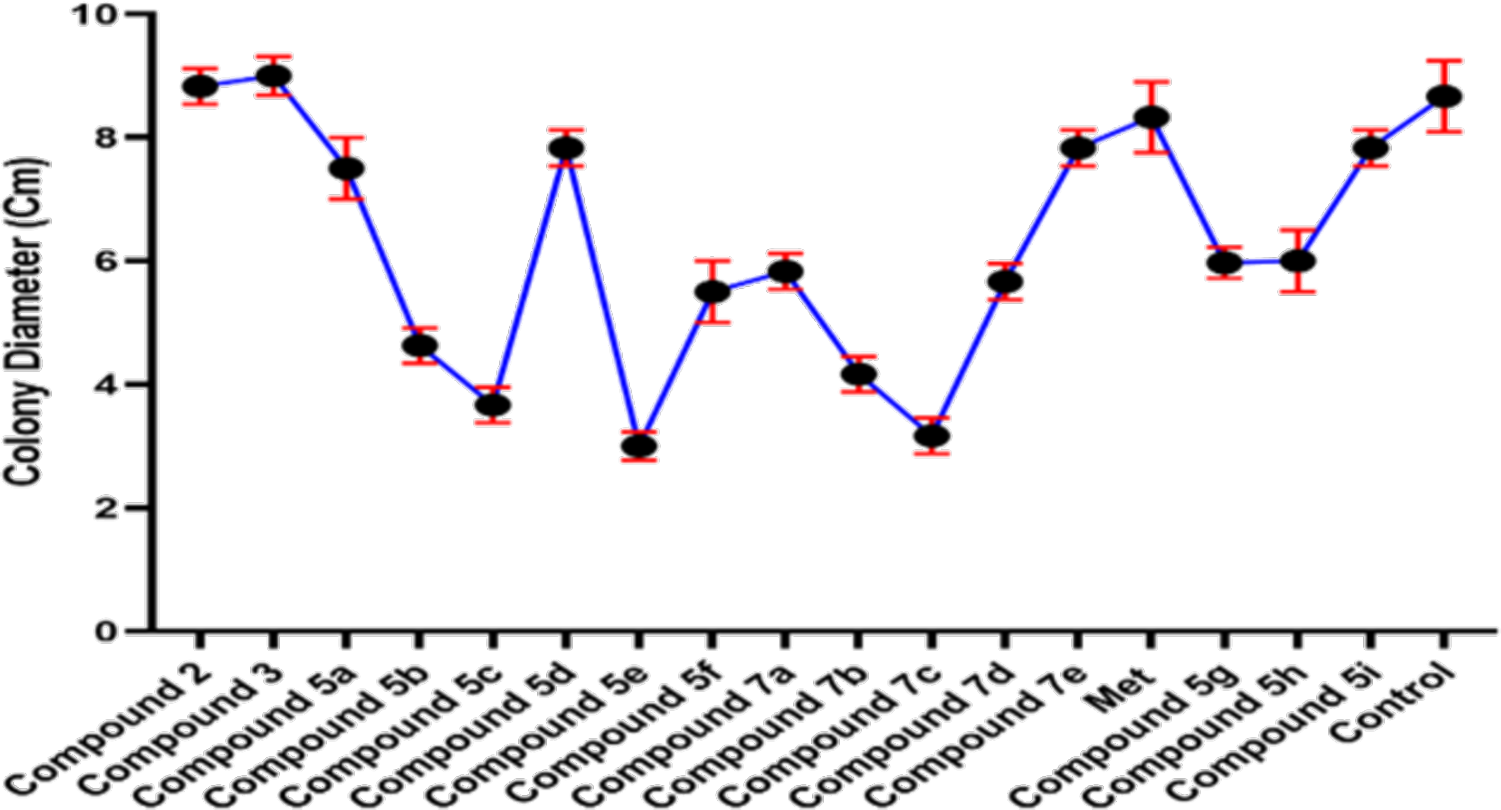
The graph representing the antifungal activity of *Didymella* sp. against compounds **5a–i** and **7a–e**.

The antifungal activity of all compounds were evaluated by inhibiting the growth of *Didymella* sp. (Figure 7 and Table 3). The fungal colony after 7 days of control treatment was noted to be 8.6 cm in diameter.

**Table 3:** Antifungal zone (cm) of metronidazole derivatives **5a–i** and **7a–e**.

Compound	Growth area in cm (diameter)			

	1st	2nd	3rd	Mean

**2**	9	9	8.5	8.833
**3**	9	9	9	9
**5a**	7	7.5	8	7.5
**5b**	5	5.5	5.5	5.33
**5c**	4	3.5	3.5	3.67
**5d**	8	7.5	8	7.83
**5e**	3	3	3	3
**5f**	5	5.5	6	5.5
**5g**	6	6	6	6.00
**5h**	5.5	6	6.5	6.00
**5i**	7.5	8	8	7.83
**7a**	6	6	5.5	5.83
**7b**	4	4.5	4	4.17
**7c**	3	3	3.5	3.167
**7d**	6	5.5	5.5	5.67
**7e**	8	8	7.5	7.83
**1**	8	9	8	8.33
control	8	9	9	8.67

Whereas, the growth of the fungal colony was detected maximum, i.e., 8.8 ± 0.2 and 9.0 ± 0.3 cm against compound **2** and **3**, respectively. However, compound **5e** and **7c** efficiently inhibited the fungal growth by limiting the colony diameter to 3 ± 0.3 and 3.1 ± 0.2 cm followed equally by compound **7b** and compound **5b** with 4.1 ± 0.3 and 4.6 ± 0.2 cm, respectively. Compared to control and metronidazole treatments, fungal growth under compound **5e** and compound **7c** treatment was detected 2.8, 2.7 folds and 2.5, 2.6 folds less, respectively. All of the synthesized compounds except compounds **2** and **3** showed a higher inhibition rate of fungal growth as compared to the control and metronidazole (Figure 7 and Table 3). The inhibition zones were recorded after 7 days of treatment and compared with growth area of fungi growing in control conditions.

### Antibacterial activity

**Figure 8:**
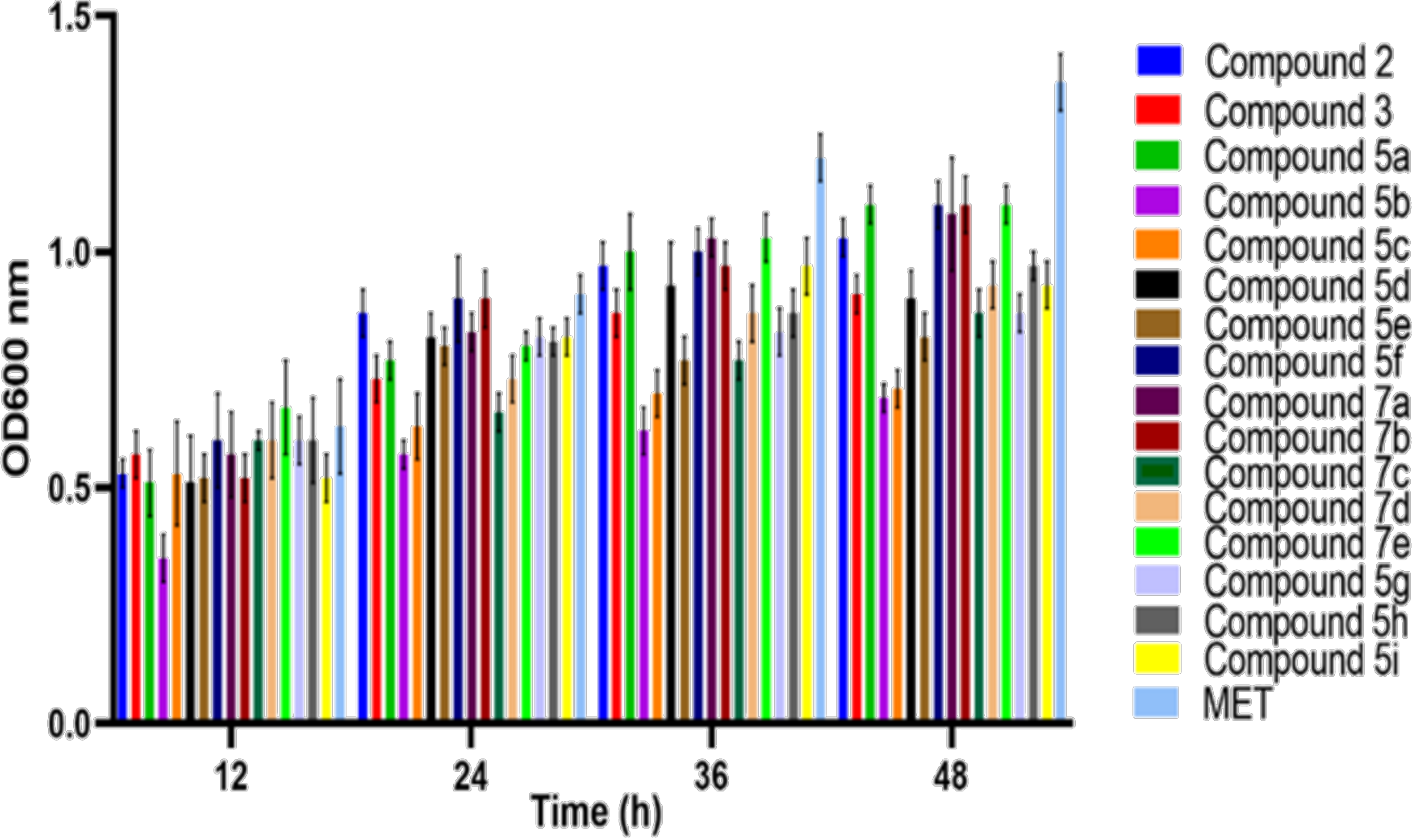
The graph representing the antibacterial activity of *E. coli* against compounds **5a–i** and **7a–e**.

To determine the bacterial growth inhibiting effects of compounds, bacterial OD_600_ was measured at different time points i.e., 12, 24, 36 and 48 h (Figure 8 and Table 4). The results revealed that all compounds were able to inhibit the bacterial growth by showing suppressed OD but with varied sensitivity. OD at 12 h reading was detected minimum, and an increase was detected over the time. At 2 h time point, the inhibitory effect of compound **5b** was significantly higher by demonstrating minimum OD among all tested compounds, while compound **7e** and metronidazole treated bacteria exhibited maximum OD. Similarly, a slight OD enhancement was recorded in bacterial growth under all tested compounds from 24–48 h. However, the trend of suppressed bacterial OD by compound **5b** was maintained at all-time points, which suggest that the inhibitory effects of compound **5b** could be sustained for a considerably longer period of time. However, inhibitory effects of compound **5c** was noted to be enhanced over the time and exhibited same inhibitory effects as compound **4** at 48 h time point. All of the tested compounds illustrated higher inhibitory effects at 36 and 48 h time point as compared to metronidazole. Taken together, the current findings demonstrate that all compounds in particular compound **5b** and **5c** inhibited bacterial growth and proved to be more potent than metronidazole.

**Table 4:** Antibacterial activities (OD 600 nm) of metronidazole derivatives **5a–i** and **7a–e**.^a^

Compound	Without compound (average)	12 h (average)	24 h (average)	36 h (average)	48 h (average)

**2**	0.370	0.530	0.701	0.870	0.971
**3**	0.400	0.570	0.622	0.731	0.870
**5a**	0.400	0.570	0.850	0.772	1.001
**5b**	0.420	0.470	0.551	0.570	0.601
**5c**	0.400	0.530	0.652	0.631	0.730
**5d**	0.370	0.530	0.801	0.872	0.931
**5e**	0.390	0.530	0.850	0.801	0.770
**5f**	0.470	0.600	0.852	0.900	1.001
**5g**	0.533	0.630	0.751	0.832	0.831
**5h**	0.433	0.600	0.903	0.831	0.870
**5i**	0.433	0.630	0.804	0.870	0.970
**7a**	0.400	0.570	0.853	0.831	1.032
**7b**	0.400	0.570	0.901	0.902	0.970
**7c**	0.400	0.600	0.602	0.670	0.770
**7d**	0.433	0.600	0.751	0.730	0.871
**7e**	0.500	0.670	0.902	0.801	1.030
**1**	0.433	0.630	0.702	0.830	1.071

^a^The bacterial growth inhibiting effects of different compounds were recorded from 12 h to 48 h. Compound **1** represents the positive control metronidazole.

## Conclusion

In summary, a series of novel metronidazole 1*H*-1,2,3-triazole and carboxylate derivatives (**5a–i** and **7a–e**) were synthesized via “click” chemistry, and evaluated for their antimicrobial activity (antifungal and antibacterial) in vitro. All the synthesized compounds (except **2** and **3** for antifungal studies) showed higher inhibition rates of fungal and bacterial growths when compared to control and the parent compound, metronidazole. Amongst the tested compounds **5b**, **5c**, **5e**, **7b** and **7e** displayed excellent potent antimicrobial activity. The present study has added one more step in exploring the 1*H*-1,2,3-triazole moiety in the medicinal field. In addition, the above-mentioned activity of all the active compounds was reported for the first time for these derivatives.

## Supporting Information

File 1 Experimental section and copies of NMR spectra.
