# Supplementary material for: Synthesis and antimicrobial activity of 1H-1,2,3-triazole and carboxylate analogues of metronidazole
Source: Beilstein J Org Chem. 2021 Sep 9;17:2377–84. doi: 10.3762/bjoc.17.154 (PMC8450958; doi:10.3762/bjoc.17.154)
Supplement: File 1 — Experimental section and copies of NMR spectra. [file Beilstein_J_Org_Chem-17-2377-s001.pdf]

BEILSTEIN JOURNAL OF ORGANIC CHEMISTRY

Supporting Information

for

Synthesis and antimicrobial activity of 1*H*-1,2,3-triazole and carboxylate analogues of metronidazole

Satya Kumar Avula, Syed Raza Shah, Khdija Al-Hosni, Muhammad U. Anwar, Rene Csuk, Biswanath Das and Ahmed Al-Harrasi

Beilstein J. Org. Chem. **2021,** *17,* 2377–2384. doi:10.3762/bjoc.17.154

Experimental section and copies of NMR spectra

License and Terms: This is a supporting information file under the terms of the Creative Commons Attribution License (https://creativecommons.org/licenses/by/4.0). Please note that the reuse, redistribution and reproduction in particular requires that the author(s) and source are credited and that individual graphics may be subject to special legal provisions.
The license is subject to the *Beilstein Journal of Organic Chemistry* terms and conditions: (https://www.beilstein-journals.org/bjoc/terms)

Contents

Experimental section

Copies of ^{1}H NMR, ^{13}C NMR, ^{19}F NMR and HRMS spectra of compounds

Experimental section

General

All experiments were carried out in dry reaction vessels under dry nitrogen atmosphere. All reagents were obtained from Sigma-Aldrich, Germany. Silica gel for column chromatography were of 100–200 mesh, Solvents were purified by following standard procedures. Optical rotations were measured using the sodium D line on a polarimeter. Thin-layer chromatography (TLC) was carried using silica gel F_{254} pre-coated plates. UV-light and I_2 stain were used to visualize the spots. The ^{1}H and ^{13}C NMR spectra were recorded on NMR spectrometer (Bruker: 600 MHz for ^{1}H, 150 MHz for ^{13}C and 564 MHz for ^{19}F) using $CDCl_3$ as a solvent. The high-resolution electrospray ionization mass spectra (HRESIMS) were recorded on an Agilent 6530 LC Q-TOF instrument. Organic extracts and solutions of pure compounds were dried over anhydrous $MgSO_4$. For X-ray measurements, single crystal of **3**, **5c, 7b,** were mounted on a MiTeGen loop with grease and examined on a Bruker D8 Venture APEX diffractometer equipped with Photon 100 CCD area detector at 296 (2) K using graphite-monochromated MoK_{α} radiation(λ = 0.71073Å). Data was collected using the APEX-II software [1], integrated using SAINT [2], and corrected for absorption using a multi-scan approach (SADABS) [3]. The structure was elucidated using intrinsic phasing (SHELXT) [4]. Final cell constants were determined from full least squares refinement of all observed reflections. All non-H atoms were located in subsequent difference maps and refined anisotropically with SHELXL-2016 [4] using full least squares refinement against F^2. H-atoms were added at calculated positions and refined with a riding model. The structure has been deposited with the CCDC (CSD deposition number = 2063128-2063130).

*2-(2-Methyl-5-nitro-1H-imidazol-1-yl)ethyl 4-methylbenzenesulfonate (**2**)*

To a solution of metronidazole (**1**, 0.8 g, 3.07 mmol) in dry DCM (25.0 mL), triethylamine (0.86 mL, 6.15 mmol) was added at 0 °C. Then tosyl chloride (0.650 g, 3.38 mmol) was added over 2 h and catalytic amount of DMAP (0.038 g, 0.30 mmol) was added. The resulting mixture was allowed to warm to room temperature and stirred for 3 h. Then the reaction mixture was treated with aqueous 1 N HCl (10 mL) and extracted with DCM (3 × 30 mL). The organic layer was washed with saturated $NaHCO_3$ (15 mL) and water (15 mL). The combined organic phases were dried over

anhydrous $MgSO_4$ and concentrated under reduced pressure. Flash chromatography of the crude product afforded metronidazoletosylate **2** [5] (1.197 g, 96%) as a yellow colour solid; m.p. 141-143 ºC; ^{1}H NMR (600 MHz, chloroform-*d*) δ 7.71 (s, 1H), 7.51 (d, *J* = 8.0 Hz, 2H), 7.21 (d, *J* = 8.0 Hz, 2H), 4.46 (t, *J* = 4.9 Hz, 2H), 4.29 (t, *J* = 4.8 Hz, 2H), 2.42 (s, 3H), 2.37 (s, 3H); ^{13}C NMR (150 MHz, chloroform-*d*) δ 151.5, 145.6, 133.1, 131.5, 130.0, 127.5, 67.6, 45.3, 39.2, 21.5, 14.4; HRMS (ESI$^+$): Found (M+H$^+$): 326.0714 $C_{13}H_{16}N_3O_5S$ required 326.0716.

*1-(2-Azidoethyl)-2-methyl-5-nitro-1H-imidazole (**3**)*

A stirred mixture of metronidazoletosylate **2** (1.000 g, 2.41 mmol) and sodium azide (0.472 g, 7.25 mmol) in DMF (30 mL) was heated for 3 h at 70 °C, cooled and then treated with ice–water (200 mL). The mixture was extracted with diethyl ether (2 × 150 mL) and the combined extracts were washed successively with water (2 × 30 mL) and brine (1 × 20 mL), dried over anhydrous $MgSO_4$, and concentrated in vacuo. The crude product was purified by column chromatography to yield the title product metroazide **3** [6] as a dark brown colour solid (0.536 g, 88%). m.p. 122-124 ºC; ^{1}H NMR (600 MHz, chloroform-*d*) δ 7.93 (s, 1H), 4.40 (t, *J* = 5.5 Hz, 2H), 3.74 (t, *J* = 5.6 Hz, 2H), 2.50 (s, 3H); ^{13}C NMR (150 MHz, chloroform-*d*) δ 151.3, 133.4, 50.9, 45.5, 14.5; HRMS (ESI$^+$): Found (M+H$^+$): 197.0740 $C_6H_9N_6O_2$ required 197.0737.

*General procedure for synthesis of 1H-1,2,3-triazole derivatives of metronidazole (**5a**–**i**)*

To a solution of metroazide **3** (1.0 equiv) and different alkyne derivatives **4a-i** (1.2 equiv) in acetonitrile (10 mL), CuI (2.0 equiv) and triethylamine (3.0 equiv) were added at room temperature, and the mixture was stirred for 3 h. The reaction mixture was diluted with EtOAc (20 mL), 10 mL of aqueous NH_4Cl was added and, the aqueous layer was extracted with EtOAc (3 × 15 mL), and the combined organic layer was washed with brine solution, dried over anhydrous $MgSO_4$, and concentrated in vacuo to obtain a crude residue that was purified by flash chromatography to obtain desired metronidazole 1*H*-1,2,3-triazole derivatives (**5a**–**i**) (85-94%).

*1-(2-(2-Methyl-5-nitro-1H-imidazol-1-yl)ethyl)-4-phenyl-1H-1,2,3-triazole (**5a**)*

Pale yellow colour solid [7]; Yield = 85%; m.p. 221-223 ºC; ^{1}H NMR (600 MHz, chloroform-*d*) δ 8.01 (s, 1H), 7.77 – 7.67 (m, 2H), 7.44 (s, 1H), 7.40 (t, *J* = 7.5

Hz, 2H), 7.33 (t, *J* = 7.5 Hz, 1H), 4.87 – 4.80 (m, *J* = 2.9 Hz, 4H), 2.01 (s, 3H); ^{13}C NMR (150 MHz, chloroform-*d*) δ 148.6, 133.7, 132.5, 129.6, 128.9, 128.6, 127.4, 125.8, 120.3, 49.6, 46.5, 13.4; HRMS (ESI$^+$): Found (M+H$^+$): 299.1153 $C_{14}H_{15}N_6O_2$ required 299.1155.

*1-(2-(2-Methyl-5-nitro-1H-imidazol-1-yl)ethyl)-4-(4-(trifluoromethyl)phenyl)-1H-1,2,3-triazole (**5b**)*

Light brown colour solid; Yield = 90%; m.p. 230-232 ºC; ^{1}H NMR (600 MHz, DMSO-*d*6) δ 8.61 (s, 1H), 8.07 (brs, 1H), 7.98 (d, *J* = 8.0 Hz, 2H), 7.78 (d, *J* = 8.0 Hz, 2H), 4.91 (t, *J* = 5.4 Hz, 2H), 4.79 (t, *J* = 5.5 Hz, 2H), 1.94 (s, 3H); ^{13}C NMR (150 MHz, DMSO-*d*6) δ 151.5, 145.2, 138.6, 134.3, 133.3, 128.3, 128.1, 125.9, 125.6, 123.5, 49.0, 46.0, 12.9; ^{19}F NMR (564 MHz, DMSO-*d*6) δ -61.19; HRMS (ESI$^+$): Found (M+H$^+$): 367.1134 $C_{15}H_{14}F_3N_6O_2$ required 367.1132.

*4-(4-Fluorophenyl)-1-(2-(2-methyl-5-nitro-1H-imidazol-1-yl)ethyl)-1H-1,2,3-triazole (**5c**)*

Light green colour solid; Yield = 92%; m.p. 224-226 ºC; ^{1}H NMR (600 MHz, DMSO-*d*6) δ 8.13 (s, 1H), 7.99 (brs, 1H), 7.67 (t, *J* = 6.7 Hz, 2H), 7.05 (d, *J* = 8.6 Hz, 2H), 4.81 (s, 2H), 4.74 (s, 2H), 1.86 (s, 3H); ^{13}C NMR (150 MHz, DMSO-*d*6) δ 163.1, 161.5, 146.5, 133.4, 127.5, 127.4, 127.0, 121.9, 115.9, 115.7, 49.3, 46.7, 13.5; ^{19}F NMR (564 MHz, DMSO-*d*6) δ -113.59; HRMS (ESI$^+$): Found (M+H$^+$): 317.1141 $C_{14}H_{14}FN_6O_2$ required 317.1143.

*Methyl 1-(2-(2-methyl-5-nitro-1H-imidazol-1-yl)ethyl)-1H-1,2,3-triazole-4-carboxylate (**5d**)*

Pale yellow colour solid [7]; Yield = 86%; m.p. 212-214 ºC; ^{1}H NMR (600 MHz, chloroform-*d*) δ 7.99 (s, 1H), 7.84 (s, 1H), 4.86 (t, *J* = 5.6 Hz, 2H), 4.82 (d, *J* = 5.6 Hz, 2H), 3.92 (s, 3H), 2.03 (s, 3H); ^{13}C NMR (150 MHz, chloroform-*d*) δ 160.4, 151.0, 140.5, 133.9, 128.2, 52.3, 49.8, 46.1, 29.6, 13.5; HRMS (ESI$^+$): Found (M+H$^+$): 281.0997 $C_{10}H_{13}N_6O_4$ required 281.0994.

*4-(4-Bromophenyl)-1-(2-(2-methyl-5-nitro-1H-imidazol-1-yl)ethyl)-1H-1,2,3-triazole (**5e**)*

Colour less solid; Yield = 89%; m.p. 235-237 ºC; ^{1}H NMR (600 MHz, DMSO-*d*6) δ 8.48 (s, 1H), 8.05 (s, 1H), 7.71 (d, *J* = 8.1 Hz, 2H), 7.64 (d, *J* = 8.1 Hz, 2H), 4.87

(t, J = 5.5 Hz, 2H), 4.75 (t, J = 5.6 Hz, 2H), 1.92 (s, 3H); ^{13}C NMR (150 MHz, DMSO-d_6) δ 151.2, 145.5, 138.4, 133.3, 131.9, 129.6, 127.1, 122.6, 121.0, 49.0, 46.0, 12.9; HRMS (ESI$^+$): Found (M+H$^+$): 377.0360 $C_{14}H_{14}{}^{79}BrN_6O_2$ required 377.0362. Found (M+H$^+$): 379.0344 $C_{14}H_{14}{}^{81}BrN_6O_2$ required 379.0341.

*4-(1-(2-(2-Methyl-5-nitro-1H-imidazol-1-yl)ethyl)-1H-1,2,3-triazol-4-yl)aniline (**5f**)*

Light brown colour solid; Yield = 87%; m.p. 240-242 ºC; ^{1}H NMR (600 MHz, chloroform-*d*) δ 7.98 (s, 1H), 7.53 – 7.47 (m, 2H), 7.28 (d, J = 1.3 Hz, 1H), 6.70 – 6.67 (m, 2H), 4.78 (s, 4H), 3.79 (s, 2H), 1.96 (d, J = 1.3 Hz, 3H); ^{13}C NMR (150 MHz, chloroform-*d*) δ 151.4, 148.9, 146.9, 133.8, 133.5, 128.6, 127.0, 120.0, 119.0, 115.1, 114.8, 49.5, 46.5, 13.3; HRMS (ESI$^+$): Found (M+H$^+$): 314.1364 $C_{14}H_{16}N_7O_2$ required 314.1367.

*1-(2-(2-methyl-5-nitro-1H-imidazol-1-yl)ethyl)-4-(p-tolyl)-1H-1,2,3-triazole (**5g**)*

Colourless solid [7]; Yield = 90%; m.p. 228-230 ºC; ^{1}H NMR (600 MHz, DMSO-d_6) δ 8.36 (s, 1H), 8.07 (s, 1H), 7.63 (d, J = 7.7 Hz, 2H), 7.23 (d, J = 7.7 Hz, 2H), 4.85 (t, J = 5.5 Hz, 2H), 4.75 (t, J = 5.6 Hz, 2H), 2.31 (s, 3H), 1.90 (s, 3H); ^{13}C NMR (150 MHz, DMSO-d_6) δ 146.7, 137.4, 133.3, 132.3, 129.5, 127.6, 125.1, 121.8, 48.9, 46.1, 20.8, 12.9; HRMS (ESI$^+$): Found (M+H$^+$): 313.1422 $C_{15}H_{17}N_6O_2$ required 313.1419.

*4-(2,4-difluorophenyl)-1-(2-(2-methyl-5-nitro-1H-imidazol-1-yl)ethyl)-1H-1,2,3-triazole (**5h**)*

Pale yellow colour solid; Yield = 94%; m.p. 236-238 ºC; ^{1}H NMR (600 MHz, chloroform-*d*) δ 7.99 (s, 2H), 7.97 (s, 1H), 7.58 (d, J = 3.0 Hz, 2H), 4.84 (t, J = 5.4 Hz, 2H), 4.81 (d, J = 4.8 Hz, 2H), 1.92 (s, 3H); ^{13}C NMR (150 MHz, chloroform-*d*) δ 160.1, 160.0, 151.2, 147.2, 141.2, 133.8, 133.6, 132.3, 128.8, 122.9, 112.2, 49.6, 46.4, 13.4-; ^{19}F NMR (564 MHz, chloroform-*d*) δ -110.74, -109.34; HRMS (ESI$^+$): Found (M+H$^+$): 335.1056 $C_{14}H_{13}F_2N_6O_2$ required 335.1058.

*4-(4-methoxyphenyl)-1-(2-(2-methyl-5-nitro-1H-imidazol-1-yl)ethyl)-1H-1,2,3-triazole (**5i**)*

Light brown colour solid [7]; Yield = 89%; m.p. 220-222 ºC; ^{1}H NMR (600 MHz, chloroform-*d*) δ 8.29 (s, 1H), 8.11 (brs, 1H), 7.66 (d, J = 8.4 Hz, 2H), 7.39 (d, J

= 8.4 Hz, 2H), 4.84 (t, *J* = 4.8 Hz, 2H), 4.74 (t, *J* = 5.4 Hz, 2H), 3.76 (s, 3H), 1.90 (s, 3H); ^{13}C NMR (150 MHz, chloroform-*d*) δ 159.6, 159.1, 146.5, 134.0, 133.2, 128.4, 126.5, 123.0, 121.3, 114.3, 55.2, 48.8, 46.2, 13.0; HRMS (ESI$^+$): Found (M+H$^+$): 329.1341 $C_{15}H_{17}N_6O_3$ required 329.1338.

*General procedure for synthesis of metronidazole carboxylate derivatives (**7a–e**)*

To a mixture of metronidazole **1** (1.0 equiv), different substituted acid chlorides **6a–e** (1.2 equiv), in the presence of pyridine (1.2 equiv) and catalytic amount of DMAP was added to dry DCM (10.0 mL). The reaction was heated at room temperature with stirring under a nitrogen atmosphere for 4–5 h. Then the reaction mixture was treated with aqueous 1 N HCl (2 × 10 mL) and extracted with DCM (3 × 30 mL). The organic layer was washed with saturated $NaHCO_3$ (15 mL) and water (15 mL). The combined organic phases were dried over anhydrous $MgSO_4$. The solvent was removed under vacuum, and the crude product was purified by silica gel column chromatography (eluting with EtOAc/hexane, 8:2) to give metronidazole carboxylate derivatives (**7a–e**) (86–93%).

*2-(2-Methyl-5-nitro-1H-imidazol-1-yl)ethyl benzoate (**7a**)*

White solid; Yield = 86%; m.p. 187-189 ºC; ^{1}H NMR (600 MHz, chloroform-*d*) δ 7.93 (s, 1H), 7.89 – 7.85 (m, 2H), 7.54 (td, *J* = 7.5, 1.4 Hz, 1H), 7.42 – 7.38 (m, 2H), 4.69 – 4.66 (m, 2H), 4.65 – 4.63 (m, 2H), 2.44 (d, *J* = 1.1 Hz, 3H); ^{13}C NMR (150 MHz, chloroform-*d*) δ 166.0, 150.8, 133.6, 133.2, 129.5, 128.9, 128.6, 62.8, 45.2, 14.3; HRMS (ESI$^+$): Found (M+H$^+$): 276.0834 $C_{13}H_{14}N_3O_4$ required 276.0836.

*2-(2-Methyl-5-nitro-1H-imidazol-1-yl)ethyl 4-nitrobenzoate (**7b**)*

Light yellow colour solid [8]; Yield = 91%; m.p. 214-216 ºC; ^{1}H NMR (600 MHz, chloroform-*d*) δ 8.26 (d, *J* = 8.5 Hz, 2H), 8.07 (d, *J* = 8.5 Hz, 2H), 7.95 (s, 1H), 4.73 (d, *J* = 4.9 Hz, 2H), 4.71 (d, *J* = 4.9 Hz, 2H), 2.48 (s, 3H); ^{13}C NMR (150 MHz, chloroform-*d*) δ 164.1, 150.8, 134.3, 133.1, 130.8, 130.6, 123.8, 123.4, 63.58, 44.9, 14.2; HRMS (ESI$^+$): Found (M+H$^+$): 321.0844 $C_{13}H_{13}N_4O_6$ required 321.0842.

*2-(2-Methyl-5-nitro-1H-imidazol-1-yl)ethyl 3,5-dinitrobenzoate (**7c**)*

Light brown colour solid; Yield = 93%; m.p. 224-226 ºC; ^{1}H NMR (600 MHz, chloroform-*d*) δ 9.21 (q, *J* = 1.9 Hz, 1H), 9.03 (t, *J* = 1.6 Hz, 2H), 7.95 (s, 1H), 4.78 (s, 4H), 2.55 (s, 3H); ^{13}C NMR (150 MHz, chloroform-*d*) δ 162.1, 148.7, 133.1, 132.5,

129.6, 129.3, 122.9, 122.0, 64.4, 44.7, 14.2; HRMS (ESI^+): Found ($M+H^+$): 366.0685 $C_{13}H_{12}N_5O_8$ required 366.0688.

*2-(2-Methyl-5-nitro-1H-imidazol-1-yl)ethyl propionate (**7d**)*

Light green colour liquid [9]; Yield = 87%; ^1H NMR (600 MHz, chloroform-*d*) δ 7.87 (d, *J* = 1.1 Hz, 1H), 4.52 (t, *J* = 5.0 Hz, 2H), 4.35 – 4.32 (m, 2H), 2.44 (s, 3H), 2.20 (td, *J* = 7.6, 1.1 Hz, 2H), 1.01 (dd, *J* = 7.6, 1.2 Hz, 3H); ^{13}C NMR (150 MHz, chloroform-*d*) δ 173.7, 150.8, 149.1, 132.8, 62.3, 45.0, 27.1, 14.1, 8.7; HRMS (ESI^+): Found ($M+H^+$): 228.0985 $C_9H_{14}N_3O_4$ required 228.0983.

*2-(2-Methyl-5-nitro-1H-imidazol-1-yl)ethyl butyrate (**7e**)*

Colourless liquid [9]; Yield = 89%; ^1H NMR (600 MHz, chloroform-*d*) δ 7.96 (s, 1H), 4.60 (t, *J* = 5.4 Hz, 2H), 4.42 (t, *J* = 4.8 Hz, 2H), 2.53 (s, 3H), 2.24 (t, *J* = 7.2 Hz, 2H), 1.59 (q, *J* = 7.2 Hz, 2H), 0.90 (t, *J* = 7.8 Hz, 3H),; ^{13}C NMR (150 MHz, chloroform-*d*) δ 172.9, 150.7, 132.9, 62.2, 45.1, 35.7, 18.1, 14.3, 13.5; HRMS (ESI^+): Found ($M+H^+$): 242.1163 $C_{10}H_{16}N_3O_4$ required 242.1165.

Antimicrobial activity

The antifungal activities of the compounds were investigated as reported by Espinel-Ingrof et al. [10]. Briefly, three sterilized filter disks (0.6 cm) of each compound (1g/mL) were kept at 3 cm around the *Didymella* sp. plug on the surface of Potato dextrose agar (PDA) plates. The fungal plug that was taken from the actively growing edge and kept mycelial side down on the plate. The plates were incubated at 28 °C after the filter disks were transferred to the inoculated PDA plates. Metronidazole was used a positive control. The growth of each *Didymella* sp. was investigated by measuring the colony diameter after 7 days. The diameter (cm) of the fungal mycelium growth was measured from the center to the edge of the colony, and each group was treated three times.

Whereas, the antibacterial activities of the compounds were evaluated by culturing *E. coli* in nutrient broth medium as reported by Bilal et al. [11]. Compounds were added to the 12 h old culture broth of bacteria with a concentration of 1 mg/mL. Thereafter, bacteria were incubated at 37 °C for 48 h and optical density (OD) was recorded after every 12h at 600 nm by xMark™ Microplate Absorbance Spectrophotometer. While, metronidazole was used as positive control.

References

1. *APEX-II,* Bruker AXS, Madison, WI, USA
2. *SAINT,* Bruker AXS, Madison, WI, USA.
3. *SADABS,* Bruker AXS, Madison, USA.
4. Sheldrick. G. M. *Acta Crystallogr., Sect. C: Struct. Chem.* **2015**, *C–71*, 3–8. doi:10.1107/S2053229614024218
5. Cao, J.; Liu, Y.; Zhang, L.; Du, F.; Ci, Y.; Zhang, Y.; Xiao, H.; Yao, X.; Shi, S.; Zhu, L.; Kung, H. F.; Qiao, J. *J. Radioanal. Nucl. Chem.* **2017,** *312,* 263–276. doi:10.1007/s10967-017-5210-5
6. Fernández, S.; Giglio, J.; Rey, A. M.; Cerecetto, H. *Bioorg. Med. Chem.* **2012,** *20,* 4040–4048. doi:10.1016/j.bmc.2012.05.010
7. Jarrad, A. M.; Karoli, T.; Debnath, A.; Tay, C. Y.; Huang, J. X.; Kaeslin, G.; Elliott, A. G.; Miyamoto, Y.; Ramu, S.; Kavanagh, A. M.; Zuegg, J.; Eckmann, L.; Blaskovich, M. A. T.; Cooper, M. A. *Eur. J. Med. Chem.* **2015,** *101,* 96–102. doi:10.1016/j.ejmech.2015.06.019
8. Salar, U.; Khan, K. M.; Taha, M.; Ismail, N. H.; Ali, B.; Qurat-ul-Ain, P.; erveen, S.; Ghufran, M.; Wadood, A. *Eur. J. Med. Chem.* **2017,** *125,* 1289–1299. doi:10.1016/j.ejmech.2016.11.031
9. Dubey, S.; Jain, V.; Precthi, G. B. *Indian J. Chem., Sect. B: Org. Chem. Incl. Med. Chem.* **2009,** *48,* 1571–1576.
10. Espinel-Ingroff, A.; Arthington-Skaggs, B.; Iqbal, N.; Ellis, D.; Pfaller, M. A.; Messer, S.; Rinaldi, M.; Fothergill, A.; Gibbs, D. L.; Wang, A. *J. Clin. Microbiol.* **2007**, *45*, 1811 LP – 1820. doi:10.1128/JCM.00134-07
11. Bilal, S.; Khan, A. L.; Shahzad, R.; Kim, Y.-H.; Imran, M.; Khan, M. J.; Al-Harrasi, A.; Kim, T. H.; Lee, I.-J. *Ecotoxicol. Environ. Saf.* **2018**, *164*, 648–658. doi:10.1016/j.ecoenv.2018.08.043

Table of Contents Page **Page**

**S1**: ^{1}H-NMR spectrum (600 MHz, $CDCl_3$) of compound **2**	S11
**S2**: ^{13}C-NMR spectrum (150 MHz, $CDCl_3$) of compound **2**	S11
**S3**: HRMS spectrum of compound **2**	S12
**S4**: ^{1}H-NMR spectrum (600 MHz, $CDCl_3$) of compound **3**	S13
**S5**: ^{13}C-NMR spectrum (150 MHz, $CDCl_3$) of compound **3**	S13
**S6**: HRMS spectrum of compound **3**	S13
**S7**: ^{1}H-NMR spectrum (600 MHz, $CDCl_3$) of compound **5a**	S14
**S8**: ^{13}C-NMR spectrum (150 MHz, $CDCl_3$) of compound **5a**	S14
**S9**: HRMS spectrum of compound **5a**	S15
**S10**: ^{1}H-NMR spectrum (600 MHz, $CDCl_3$) of compound **5b**	S15
**S11**: ^{13}C-NMR spectrum (150 MHz, $CDCl_3$) of compound **5b**	S16
**S12**: ^{19}F-NMR spectrum (564 MHz, $CDCl_3$) of compound **5b**	S16
**S13**: HRMS spectrum of compound **5b**	S17
**S14**: ^{1}H-NMR spectrum (600 MHz, $CDCl_3$) of compound **5c**	S17
**S15**: ^{13}C-NMR spectrum (150 MHz, $CDCl_3$) of compound **5c**	S18
**S16**: ^{19}F-NMR spectrum (564 MHz, $CDCl_3$) of compound **5c**	S18
**S17**: HRMS spectrum of compound **5c**	S19
**S18**: ^{1}H-NMR spectrum (600 MHz, $CDCl_3$) of compound **5d**	S20
**S19**: ^{13}C-NMR spectrum (150 MHz, $CDCl_3$) of compound **5d**	S20
**S20**: HRMS spectrum of compound **5d**	S20
**S21**: ^{1}H-NMR spectrum (600 MHz, $CDCl_3$) of compound **5e**	S21
**S22**: ^{13}C-NMR spectrum (150 MHz, $CDCl_3$) of compound **5e**	S21
**S23**: HRMS spectrum of compound **5e**	S22
**S24**: ^{1}H-NMR spectrum (600 MHz, $CDCl_3$) of compound **5f**	S22
**S25**: ^{13}C-NMR spectrum (150 MHz, $CDCl_3$) of compound **5f**	S23
**S26**: HRMS spectrum of compound **5f**	S23
**S27**: ^{1}H-NMR spectrum (600 MHz, $CDCl_3$) of compound **5g**	S24
**S28**: ^{13}C-NMR spectrum (150 MHz, $CDCl_3$) of compound **5g**	S24
**S29**: HRMS spectrum of compound **5g**	S25
**S30**: ^{1}H-NMR spectrum (600 MHz, $CDCl_3$) of compound **5h**	S25
**S31**: ^{13}C-NMR spectrum (150 MHz, $CDCl_3$) of compound **5h**	S26

**S32**: ^{19}F-NMR spectrum (564 MHz, $CDCl_3$) of compound **5h**	S26
**S33**: HRMS spectrum of compound **5h**	S27
**S34**: ^{1}H-NMR spectrum (600 MHz, $CDCl_3$) of compound **5i**	S27
**S35**: ^{13}C-NMR spectrum (150 MHz, $CDCl_3$) of compound **5i**	S28
**S36**: HRMS spectrum of compound **5i**	S28
**S37**: ^{1}H-NMR spectrum (600 MHz, $CDCl_3$) of compound **7a**	S29
**S38**: ^{13}C-NMR spectrum (150 MHz, $CDCl_3$) of compound **7a**	S29
**S39**: HRMS spectrum of compound **7a**	S30
**S40**: ^{1}H-NMR spectrum (600 MHz, $CDCl_3$) of compound **7b**	S30
**S41**: ^{13}C-NMR spectrum (150 MHz, $CDCl_3$) of compound **7b**	S31
**S42**: HRMS spectrum of compound **7b**	S31
**S43**: ^{1}H-NMR spectrum (600 MHz, $CDCl_3$) of compound **7c**	S32
**S44**: ^{13}C-NMR spectrum (150 MHz, $CDCl_3$) of compound **7c**	S32
**S45**: HRMS spectrum of compound 7**c**	S33
**S46**: ^{1}H-NMR spectrum (600 MHz, $CDCl_3$) of compound **7d**	S33
**S47**: ^{13}C-NMR spectrum (150 MHz, $CDCl_3$) of compound **7d**	S34
**S48**: HRMS spectrum of compound **7d**	S34
**S49**: ^{1}H-NMR spectrum (600 MHz, $CDCl_3$) of compound **7e**	S35
**S50**: ^{13}C-NMR spectrum (150 MHz, $CDCl_3$) of compound **7e**	S35
**S51**: HRMS spectrum of compound **7e**	S36

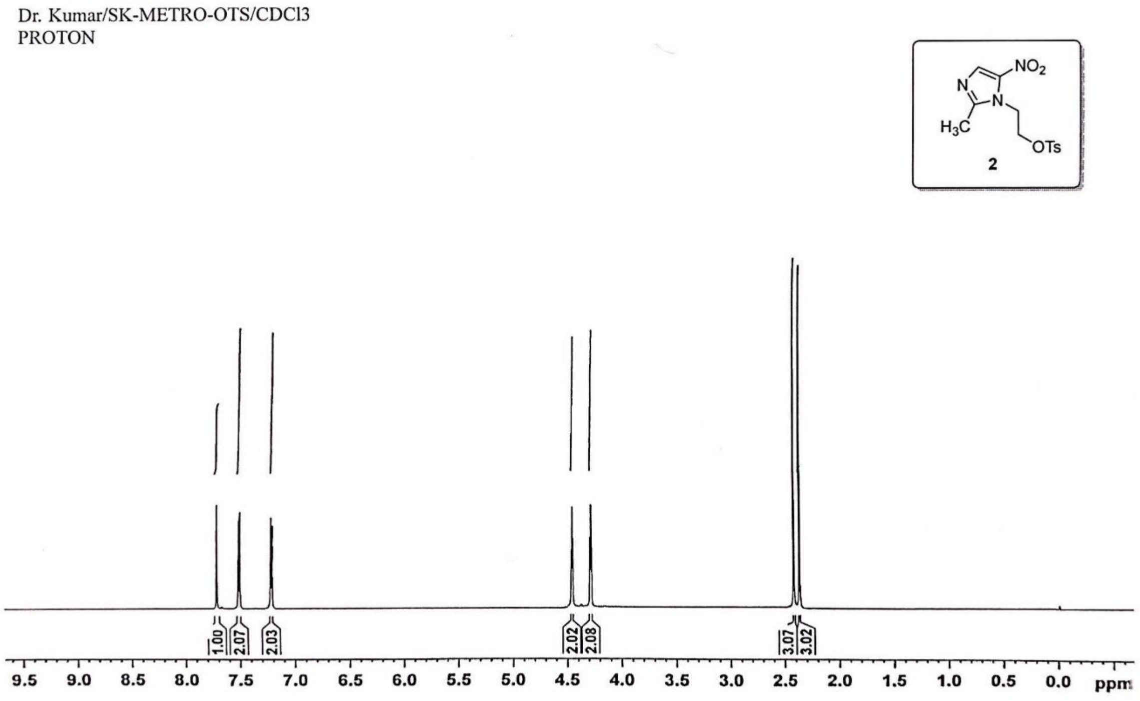

S1: ^{1}H-NMR spectrum (600 MHz, $CDCl_3$) of compound **2**

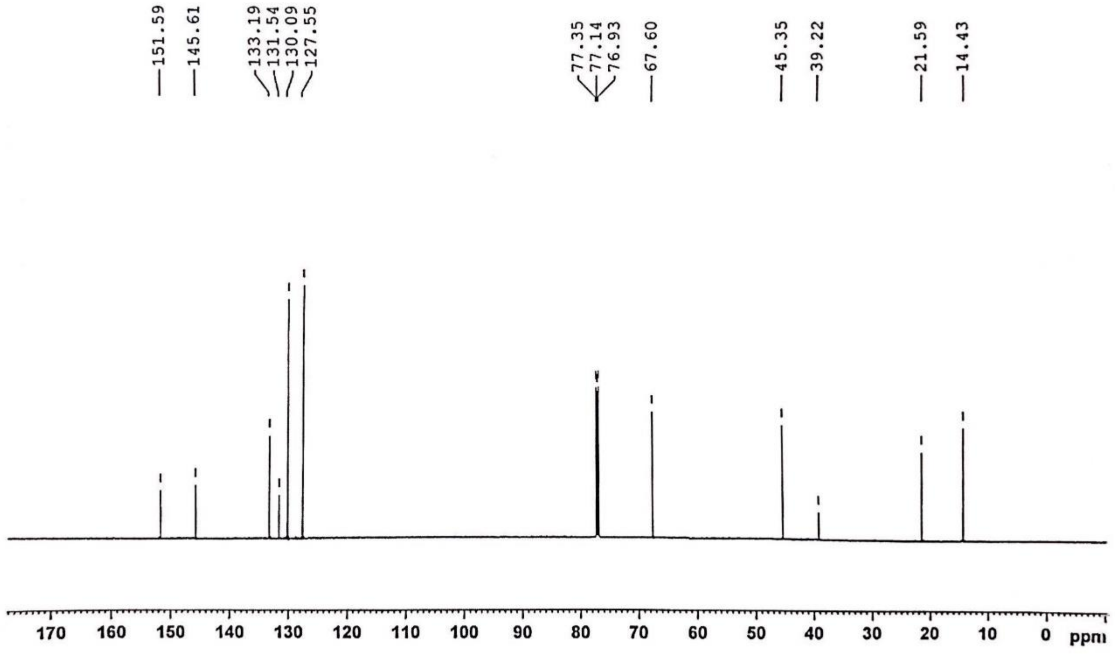

S2: ^{13}C-NMR spectrum (150 MHz, $CDCl_3$) of compound **2**

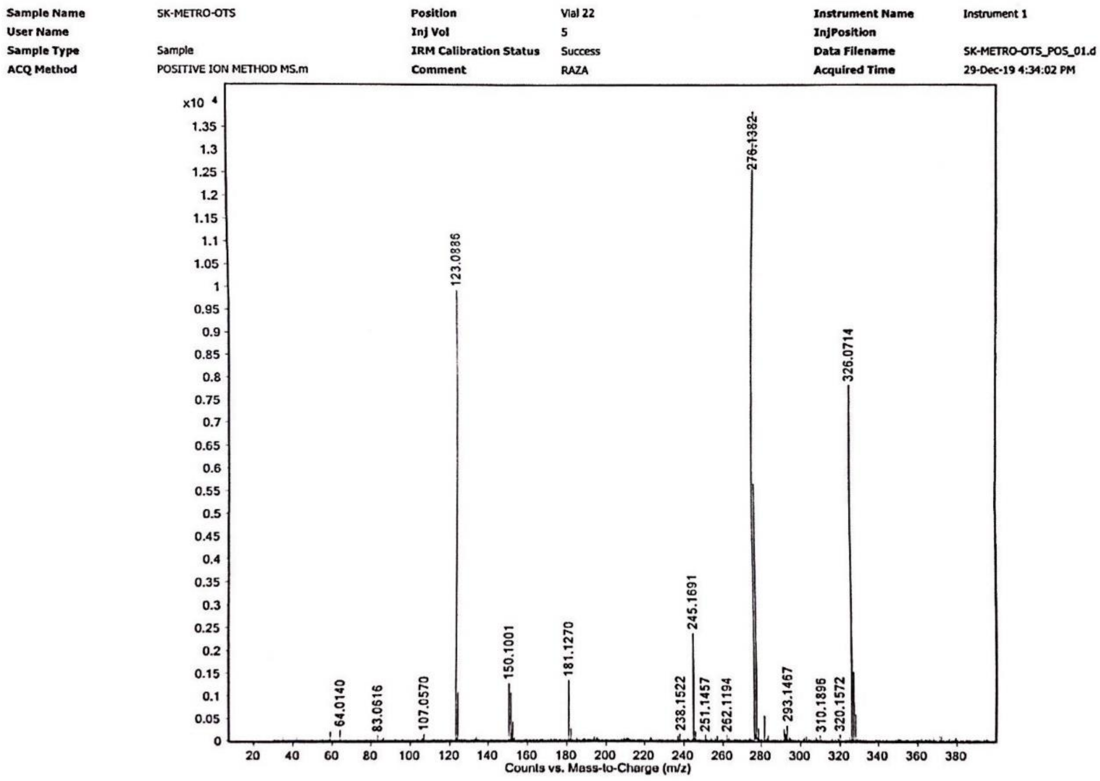

S3: HRMS spectrum of compound **2**

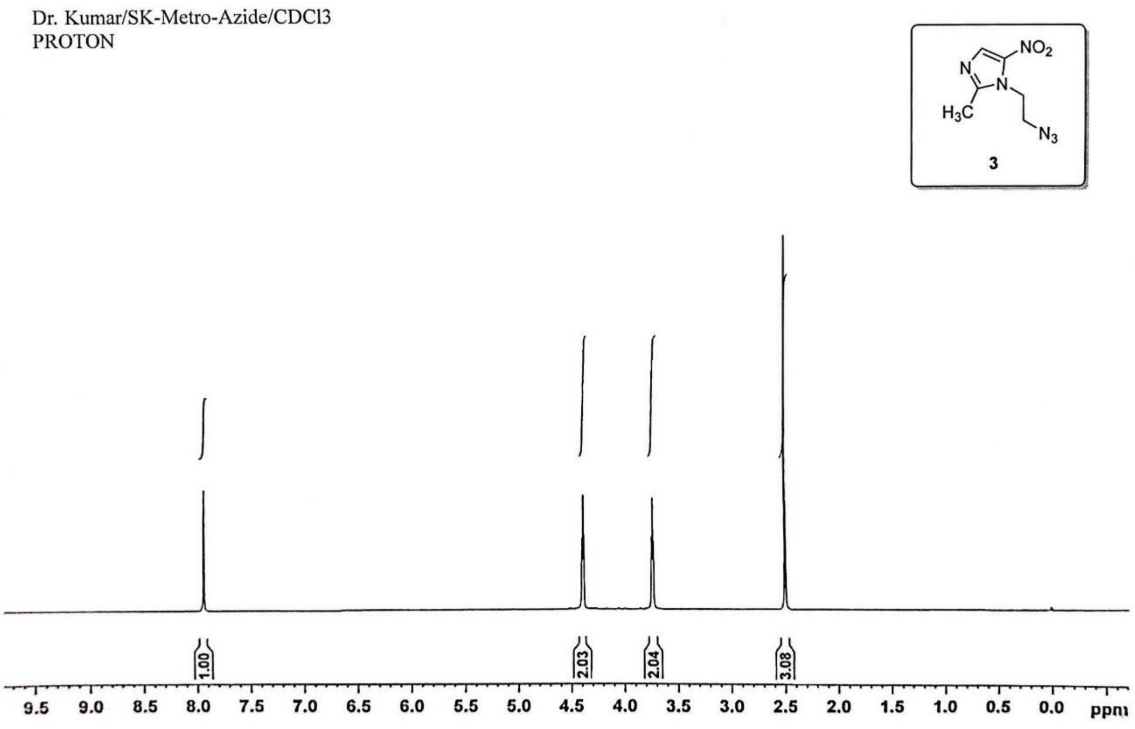

S4: ^{1}H-NMR spectrum (600 MHz, $CDCl_3$) of compound **3**

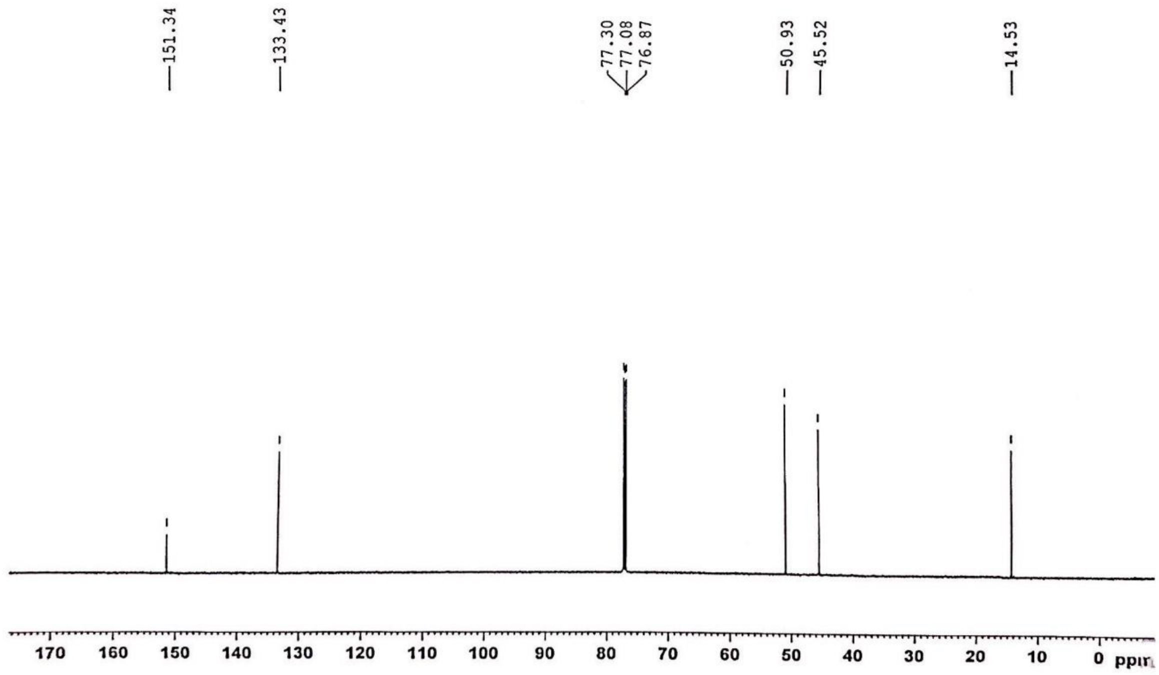

S5: ^{13}C-NMR spectrum (150 MHz, $CDCl_3$) of compound **3**

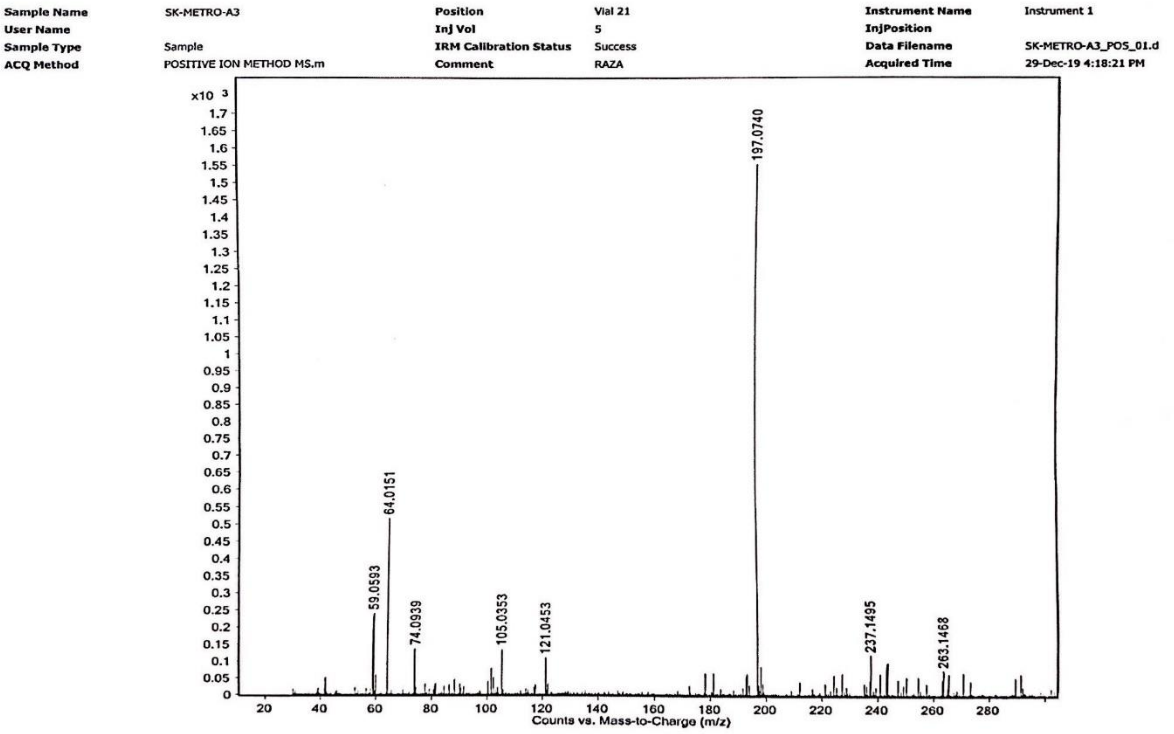

S6: HRMS spectrum of compound **3**

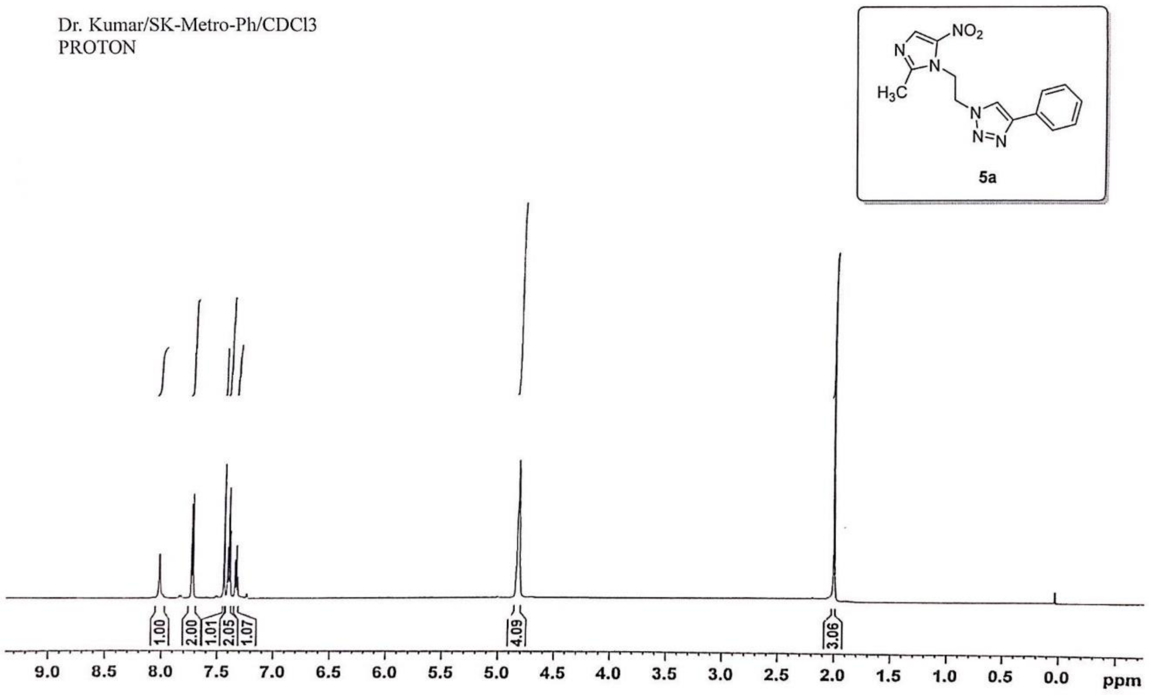

S7: ^{1}H-NMR spectrum (600 MHz, $CDCl_3$) of compound **5a**

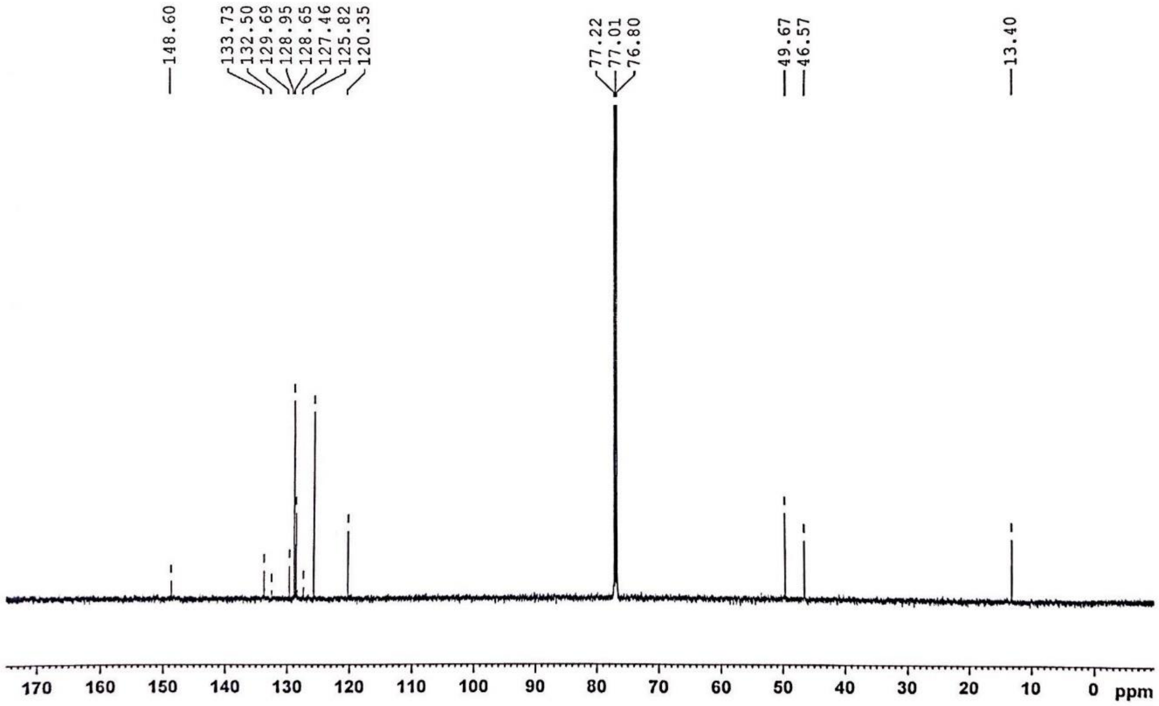

S8: ^{13}C-NMR spectrum (150 MHz, $CDCl_3$) of compound **5a**

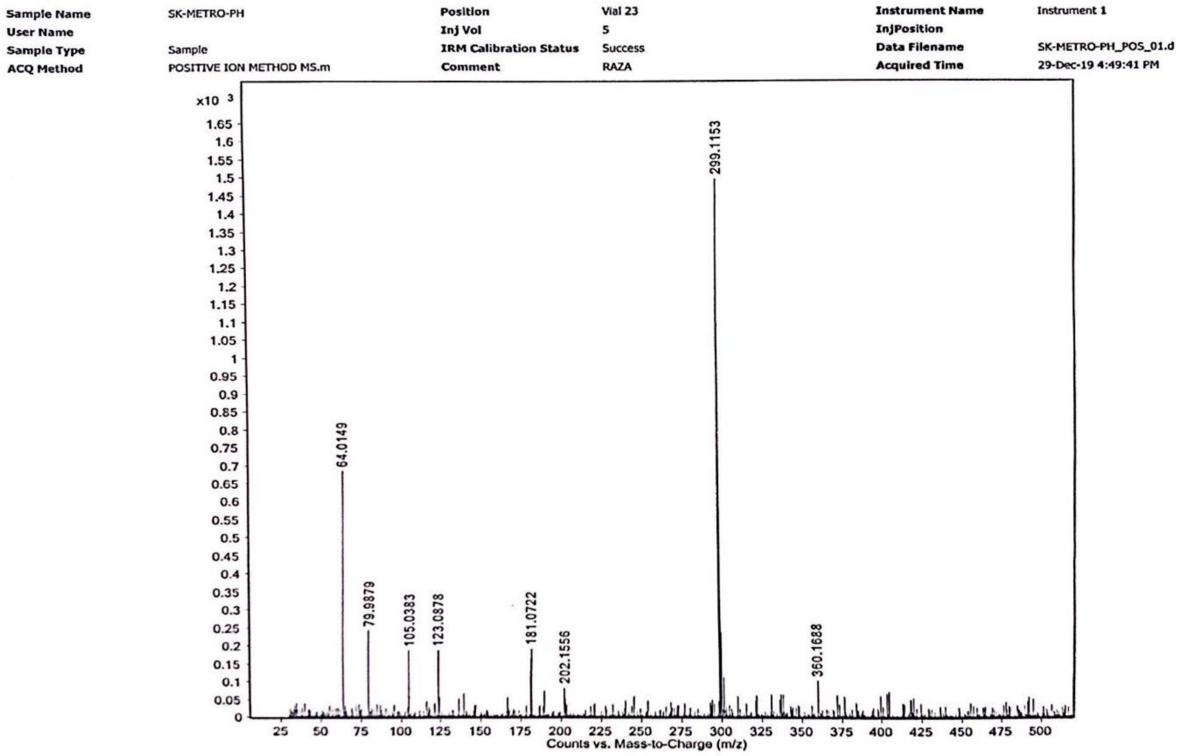

S9: HRMS spectrum of compound **5a**

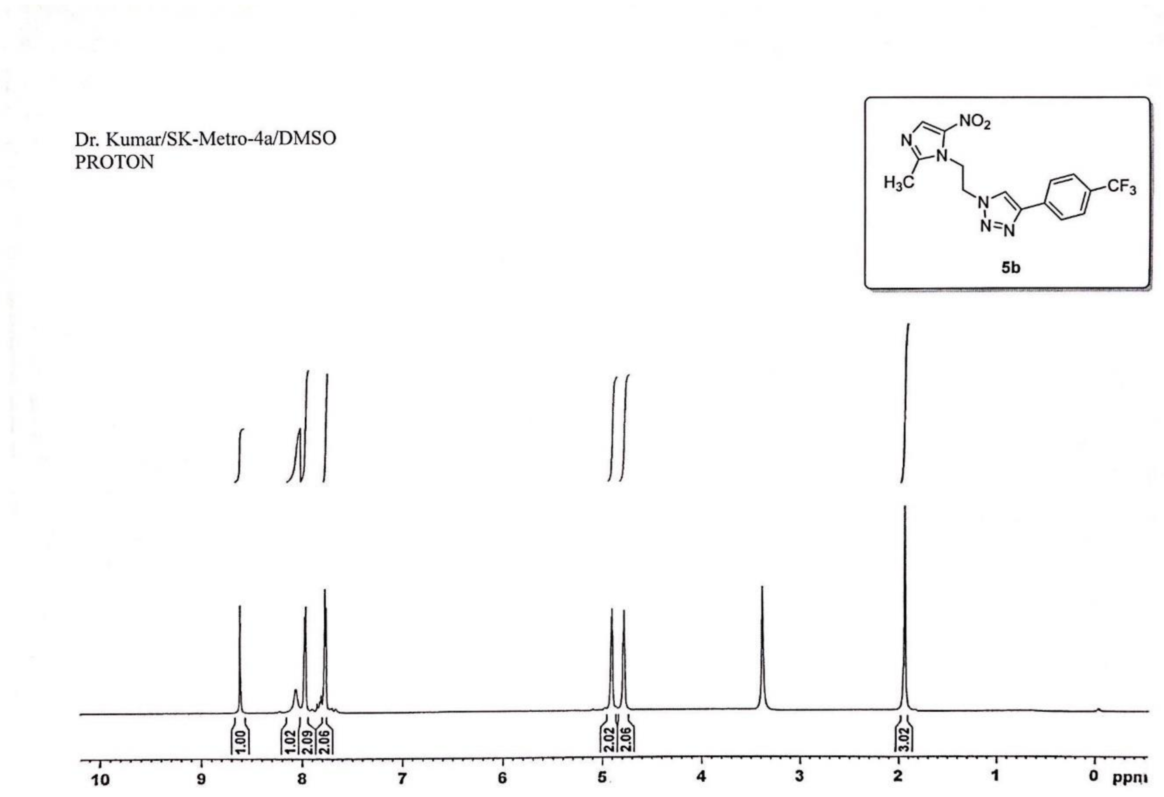

S10: ^{1}H-NMR spectrum (600 MHz, $CDCl_3$) of compound **5b**

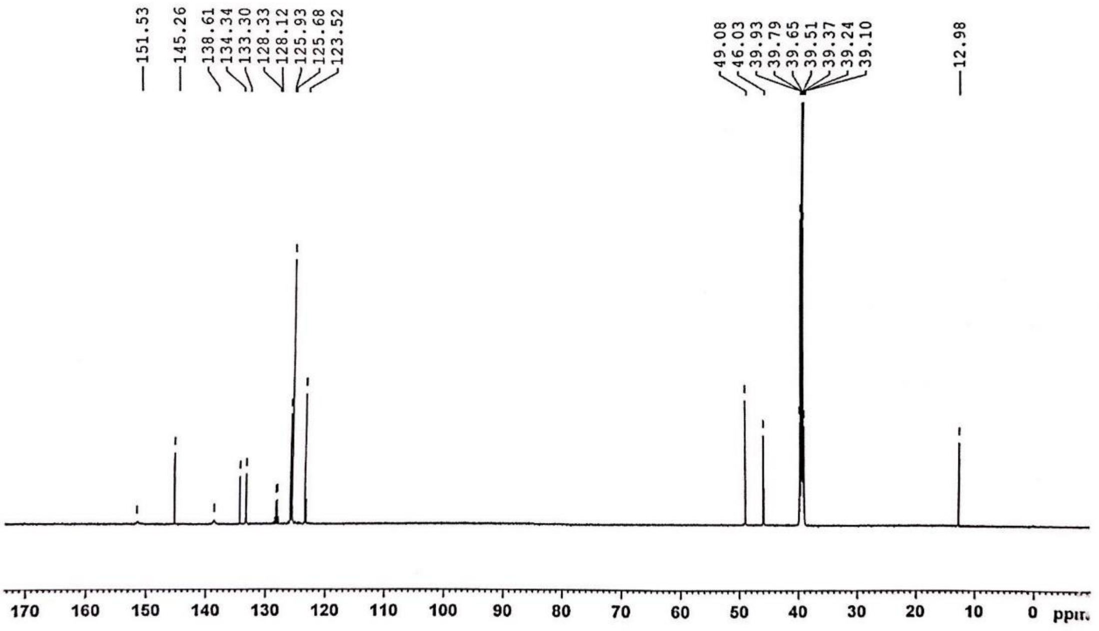

S11: ^{13}C-NMR spectrum (150 MHz, $CDCl_3$) of compound **5b**

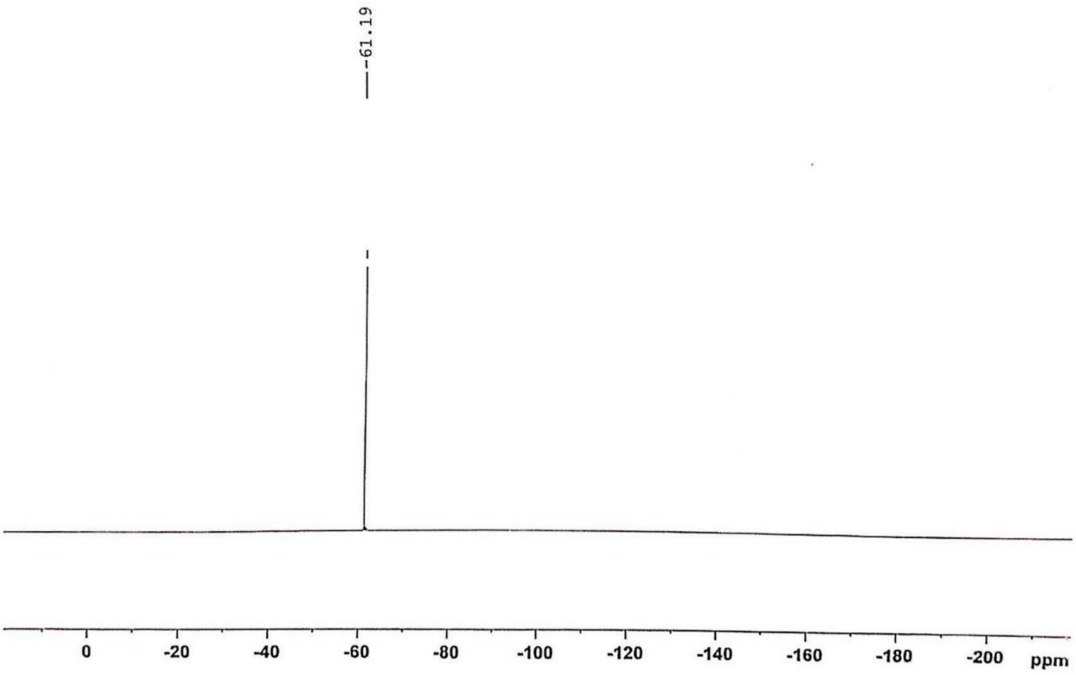

S12: ^{19}F-NMR spectrum (564 MHz, $CDCl_3$) of compound **5b**

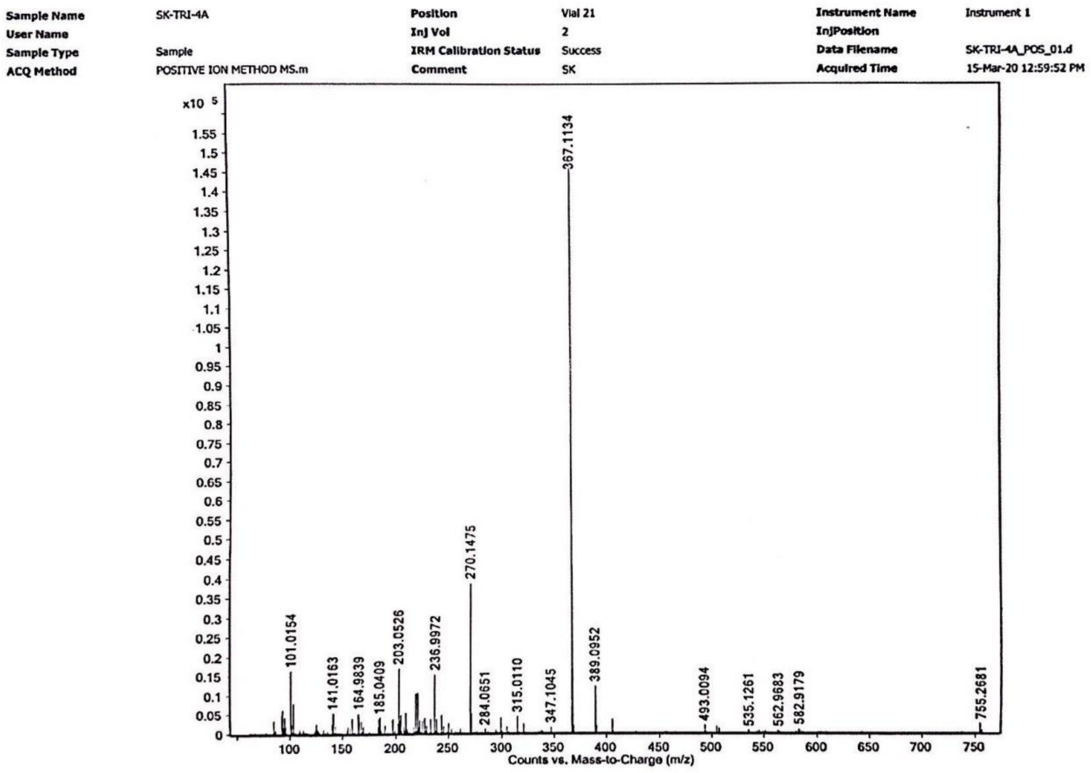

S13: HRMS spectrum of compound **5b**

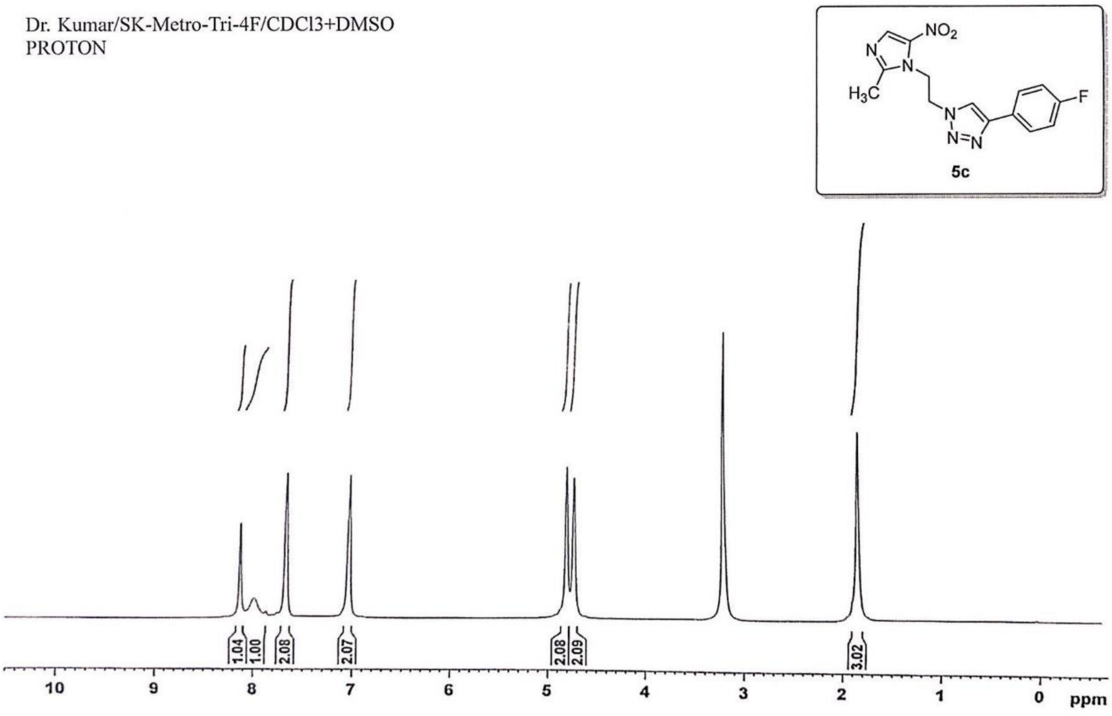

S14: ^{1}H-NMR spectrum (600 MHz, $CDCl_3$) of compound **5c**

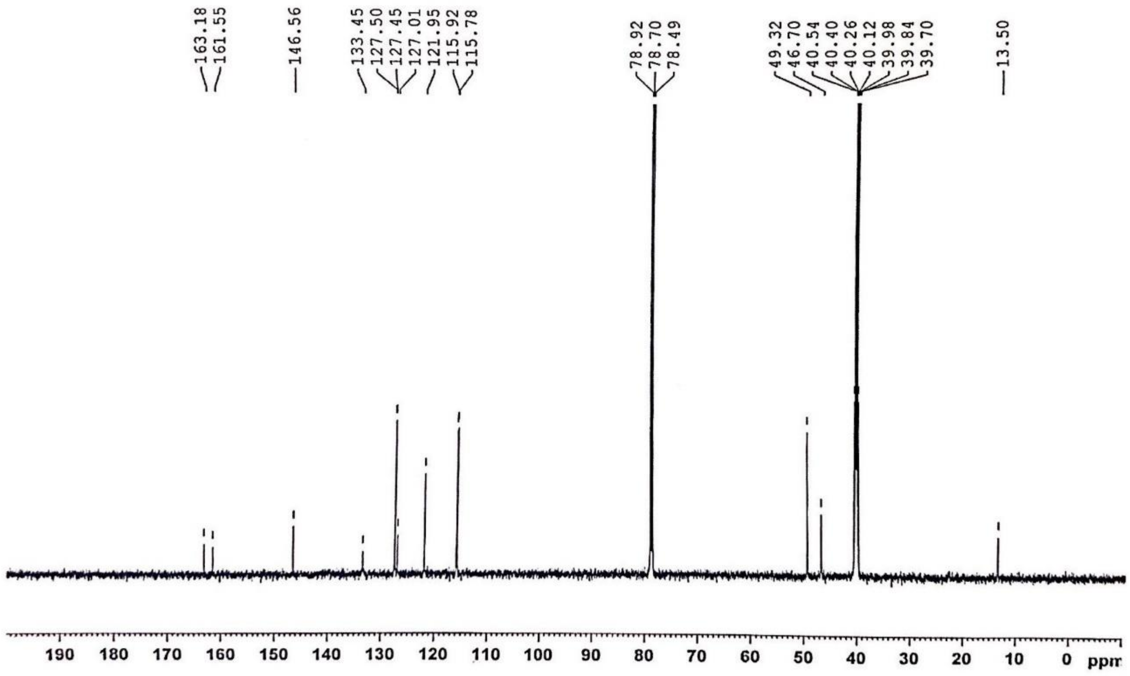

S15: ^{13}C-NMR spectrum (150 MHz, $CDCl_3$) of compound **5c**

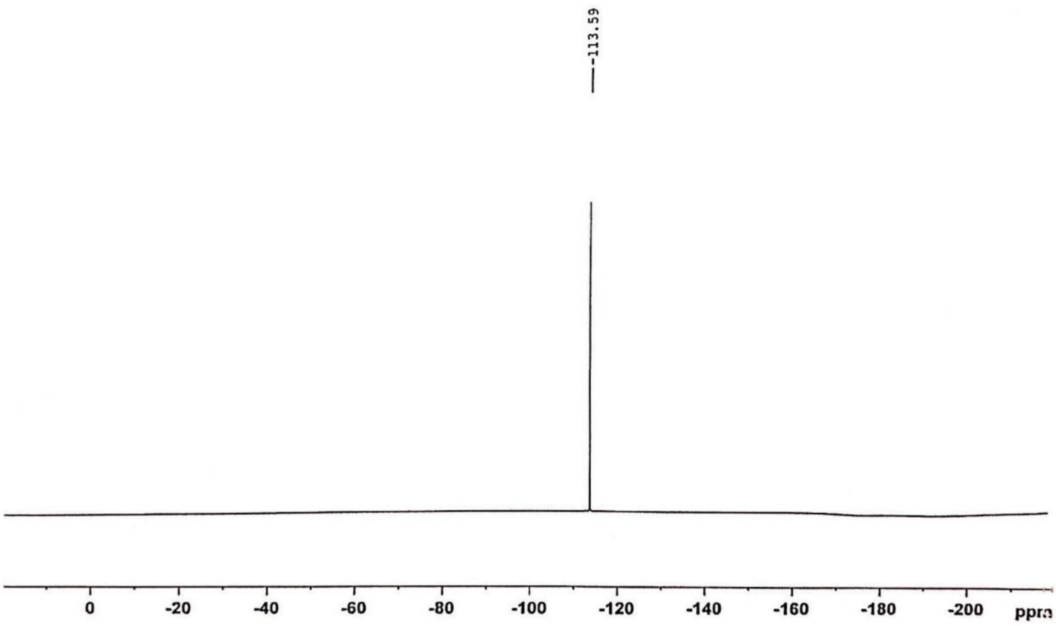

S16: ^{19}F-NMR spectrum (564 MHz, $CDCl_3$) of compound **5c**

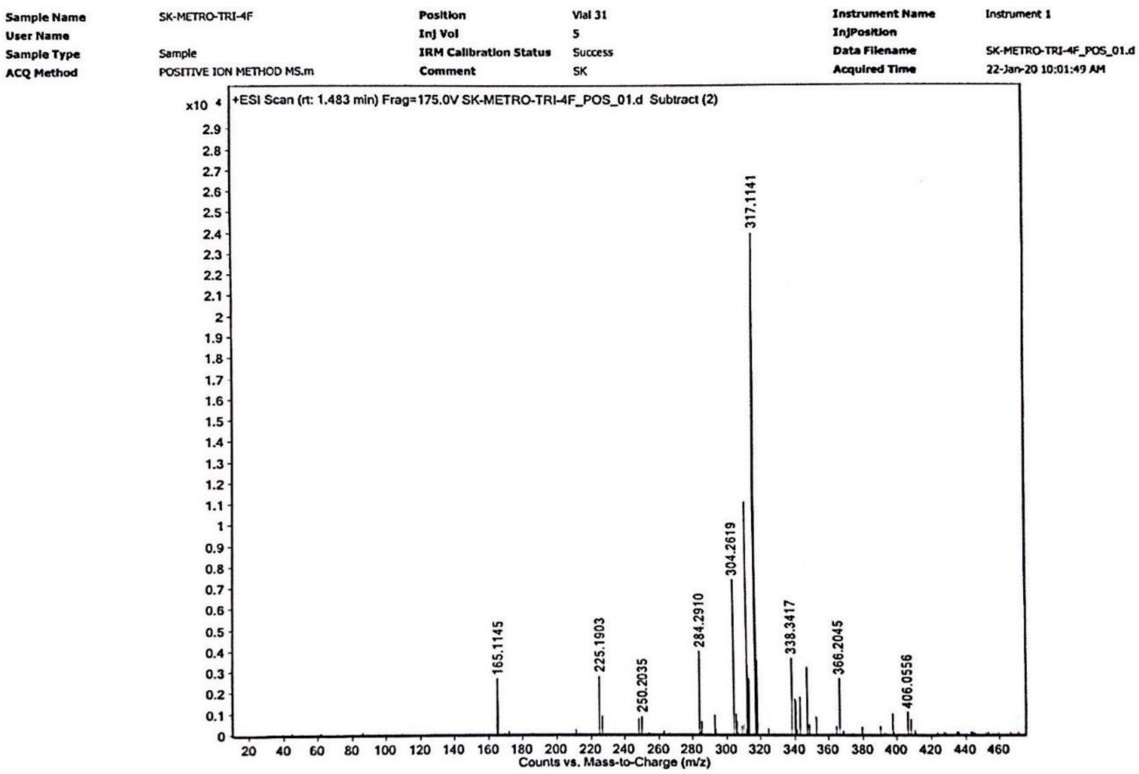

S17: HRMS spectrum of compound **5c**

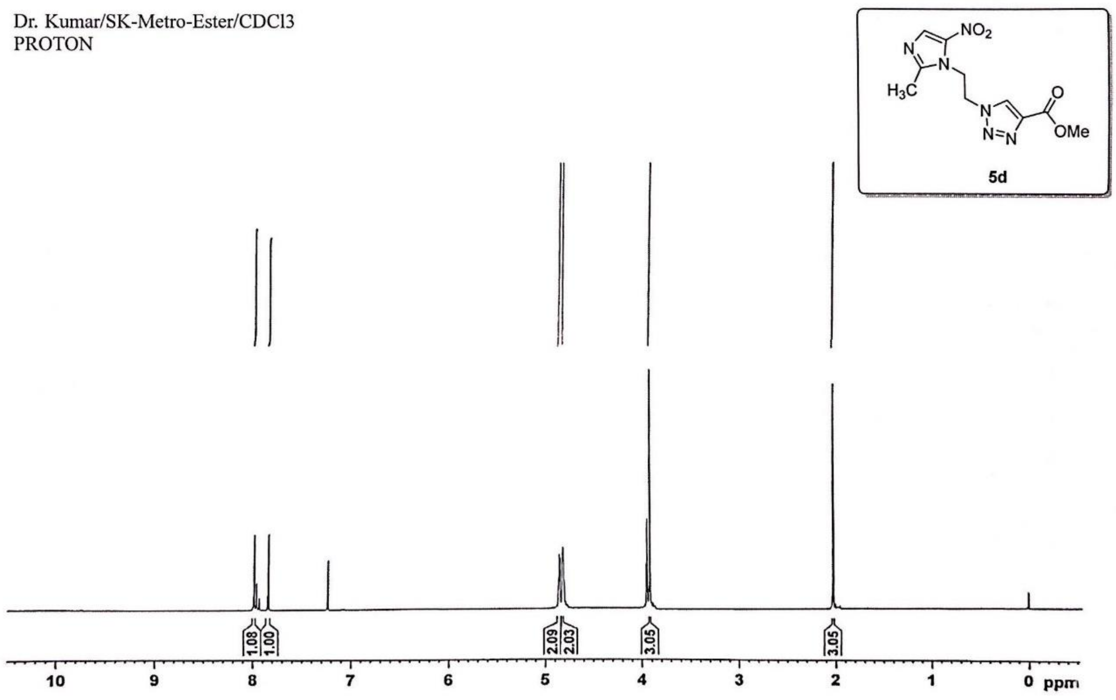

18: ^{1}H-NMR spectrum (600 MHz, $CDCl_3$) of compound **5d**

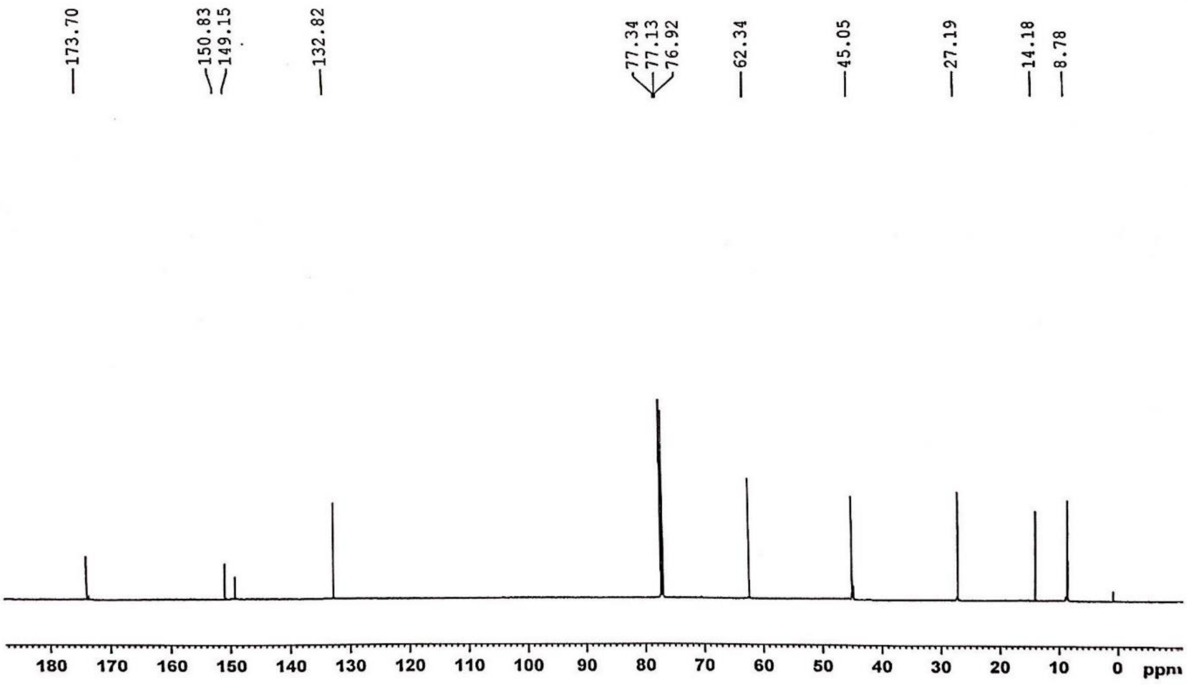

S19: ^{13}C-NMR spectrum (150 MHz, $CDCl_3$) of compound **5d**

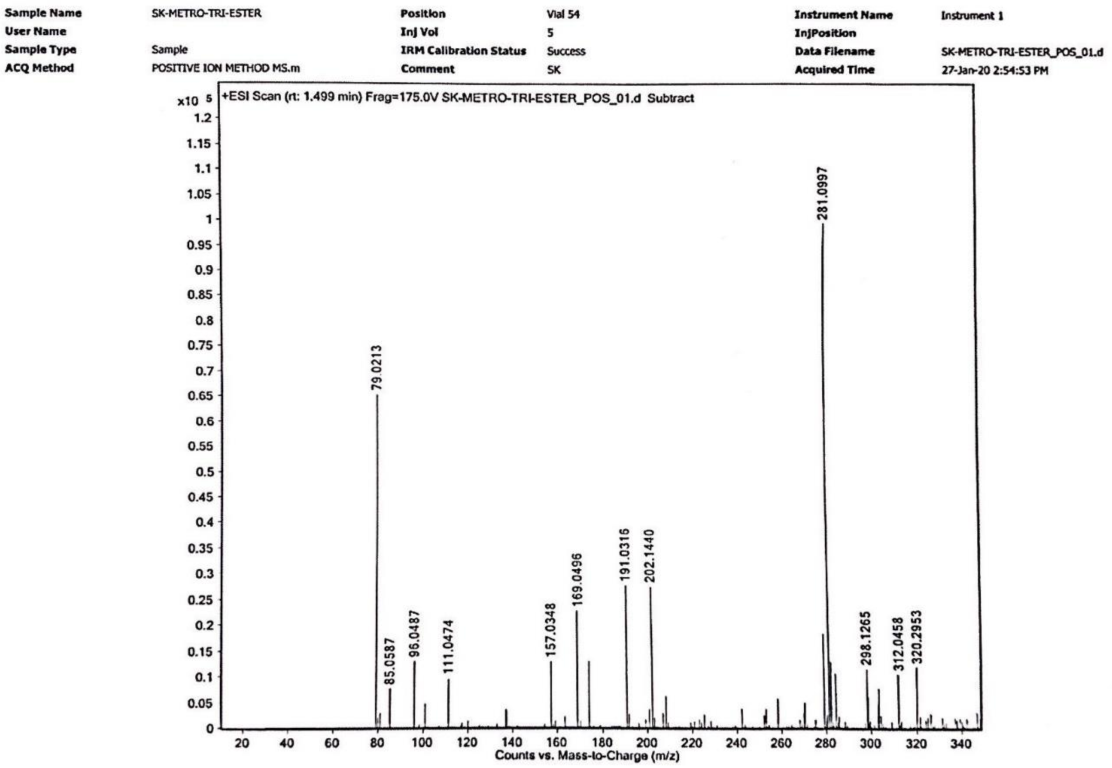

S20: HRMS spectrum of compound **5d**

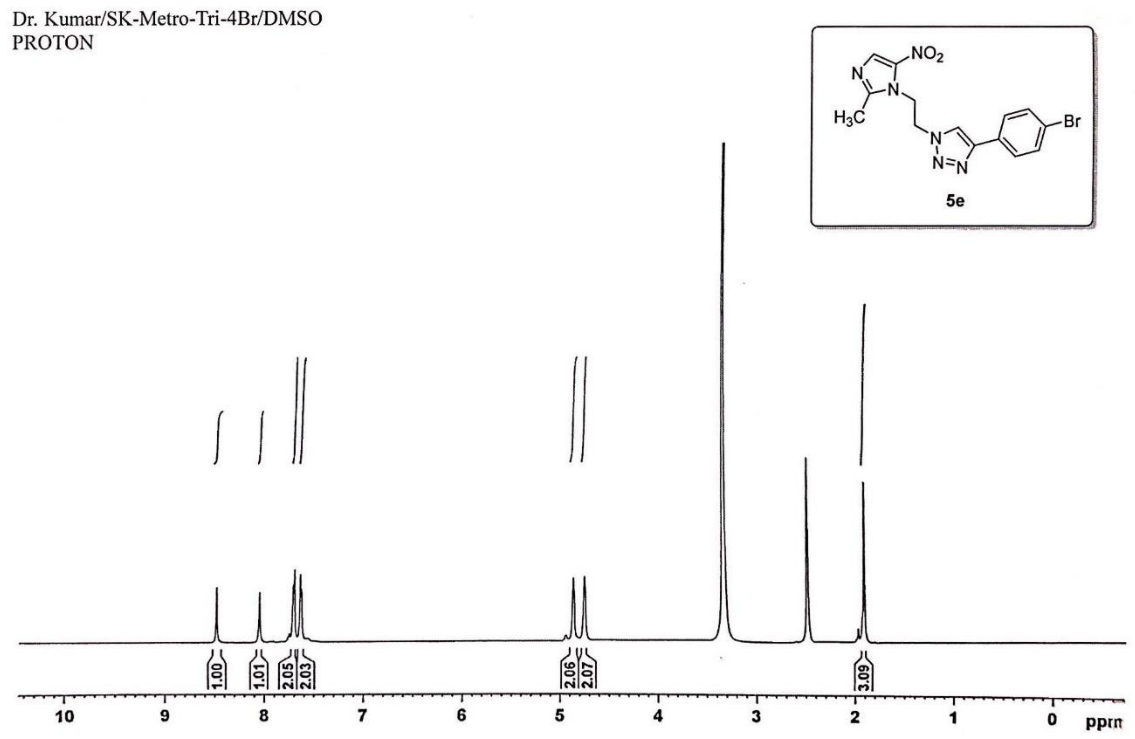

S21: ^{1}H-NMR spectrum (600 MHz, $CDCl_3$) of compound **5e**

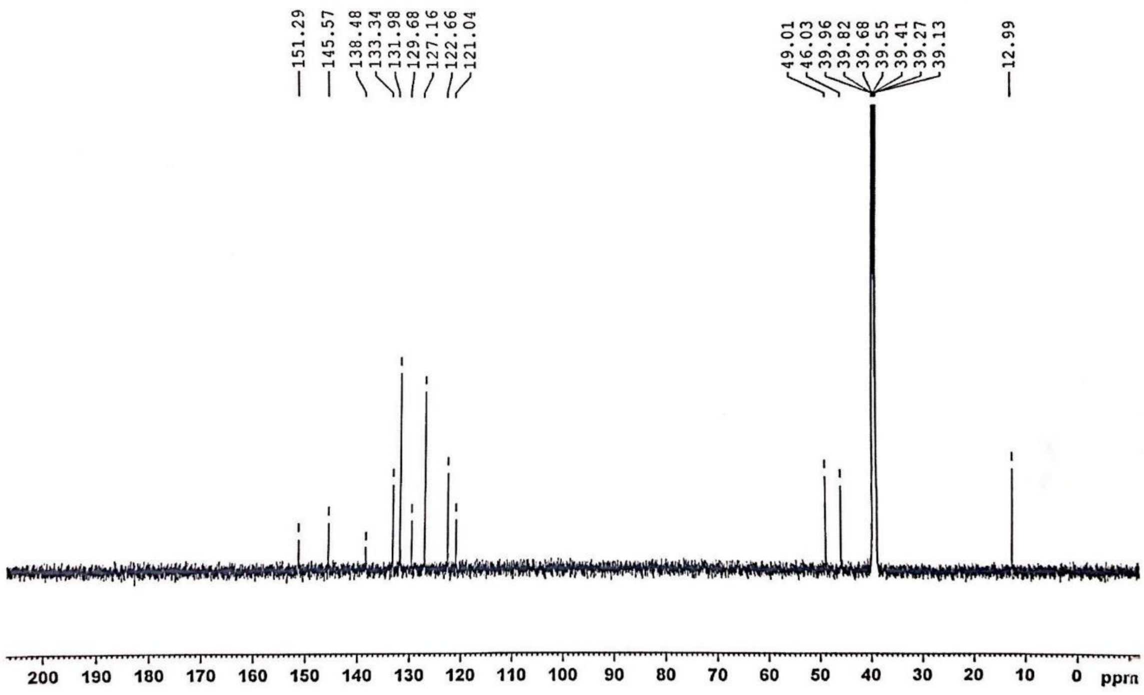

S22: ^{13}C-NMR spectrum (150 MHz, $CDCl_3$) of compound **5e**

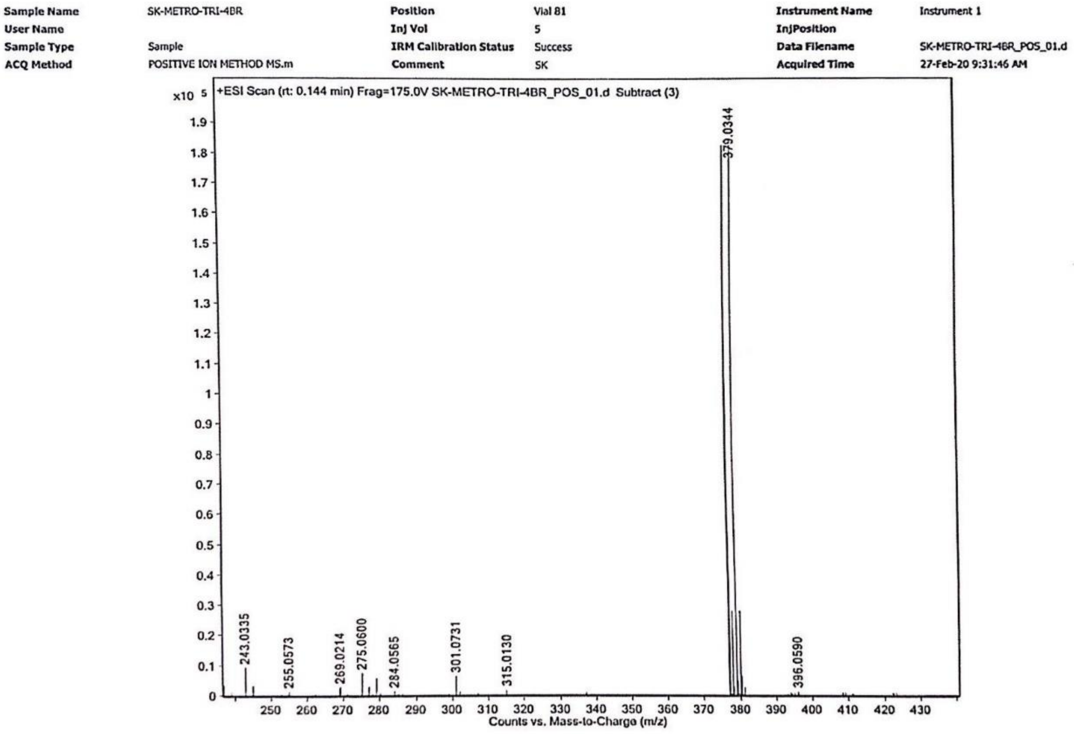

S23: HRMS spectrum of compound **5e**

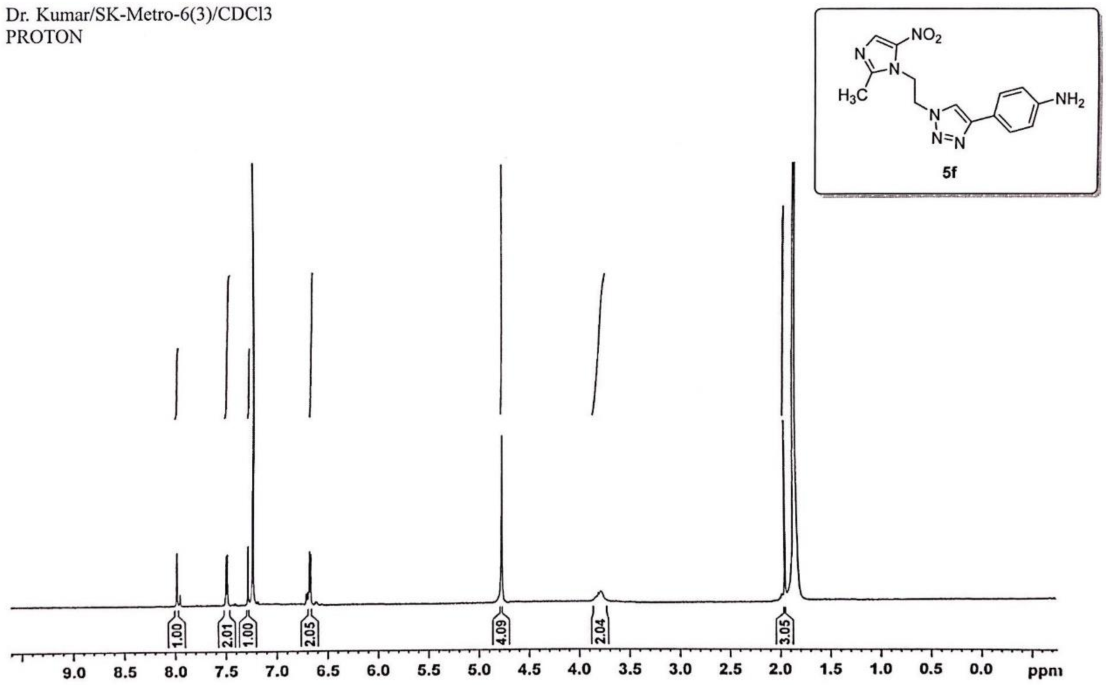

S24: ^{1}H-NMR spectrum (600 MHz, $CDCl_3$) of compound **5f**

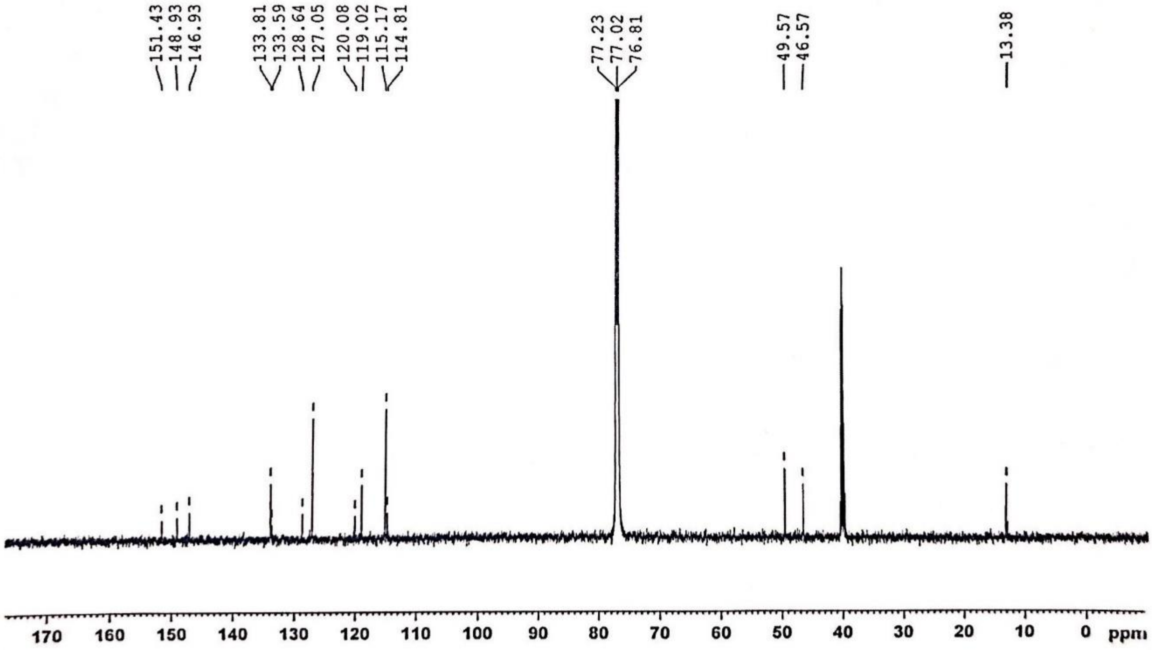

S25: ^{13}C-NMR spectrum (150 MHz, $CDCl_3$) of compound **5f**

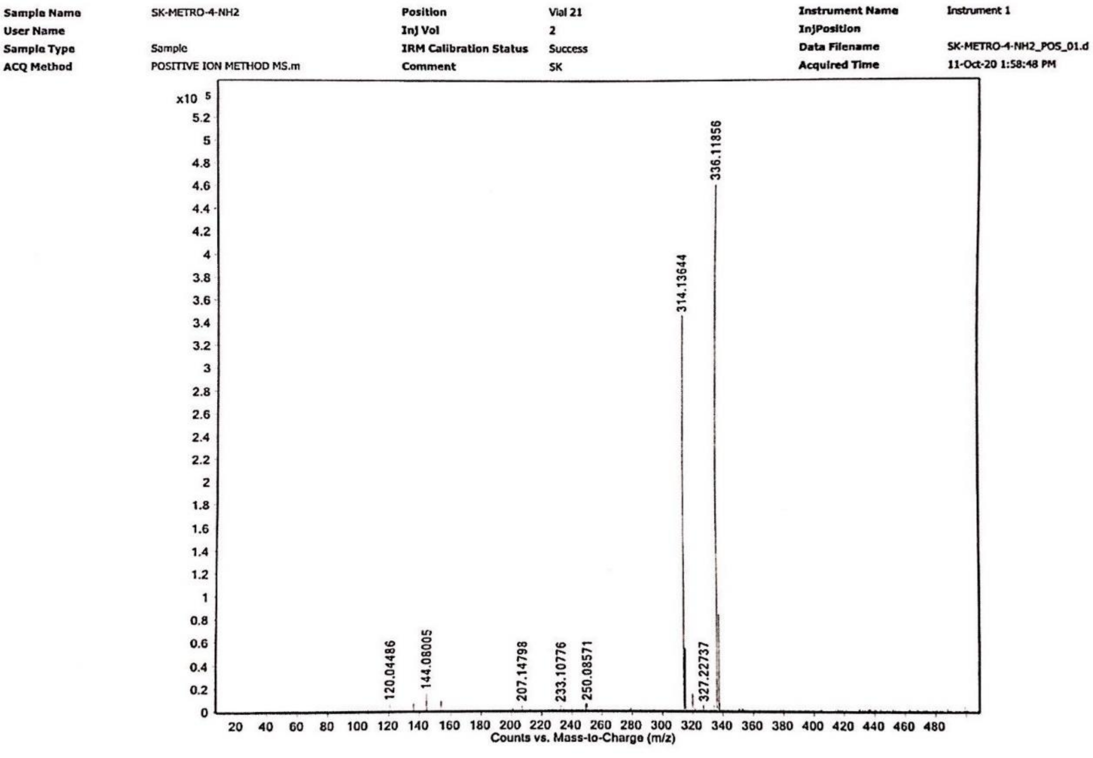

S26: HRMS spectrum of compound **5f**

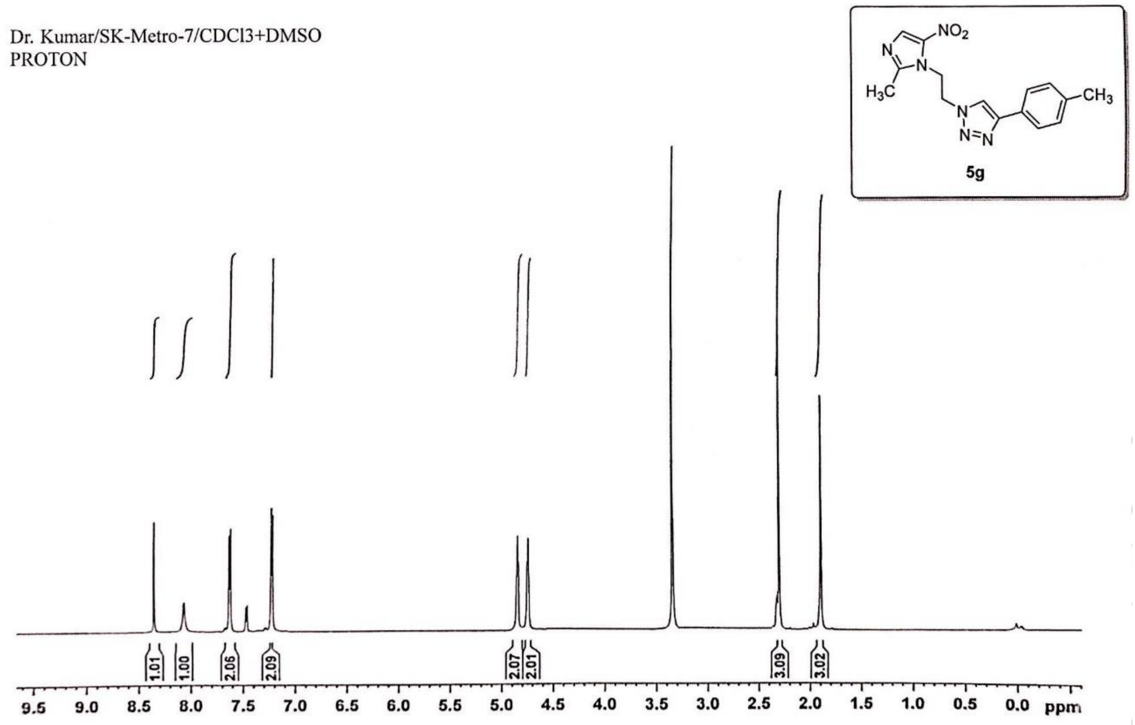

S27: ^{1}H-NMR spectrum (600 MHz, $CDCl_3$) of compound **5g**

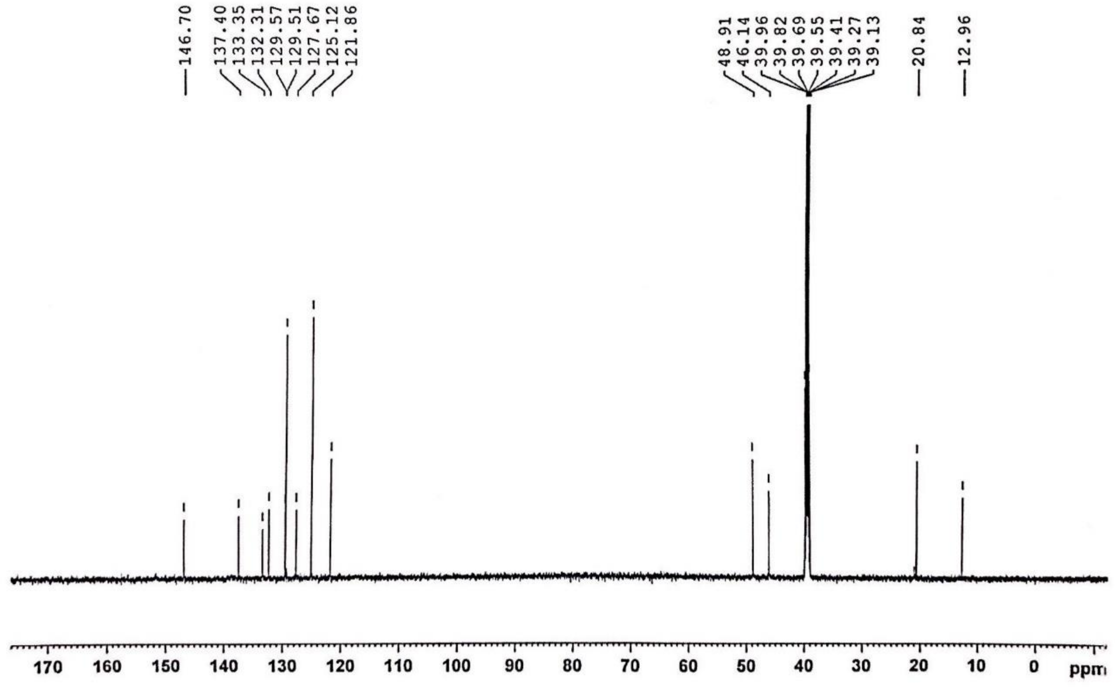

S28: ^{13}C-NMR spectrum (150 MHz, $CDCl_3$) of compound **5g**

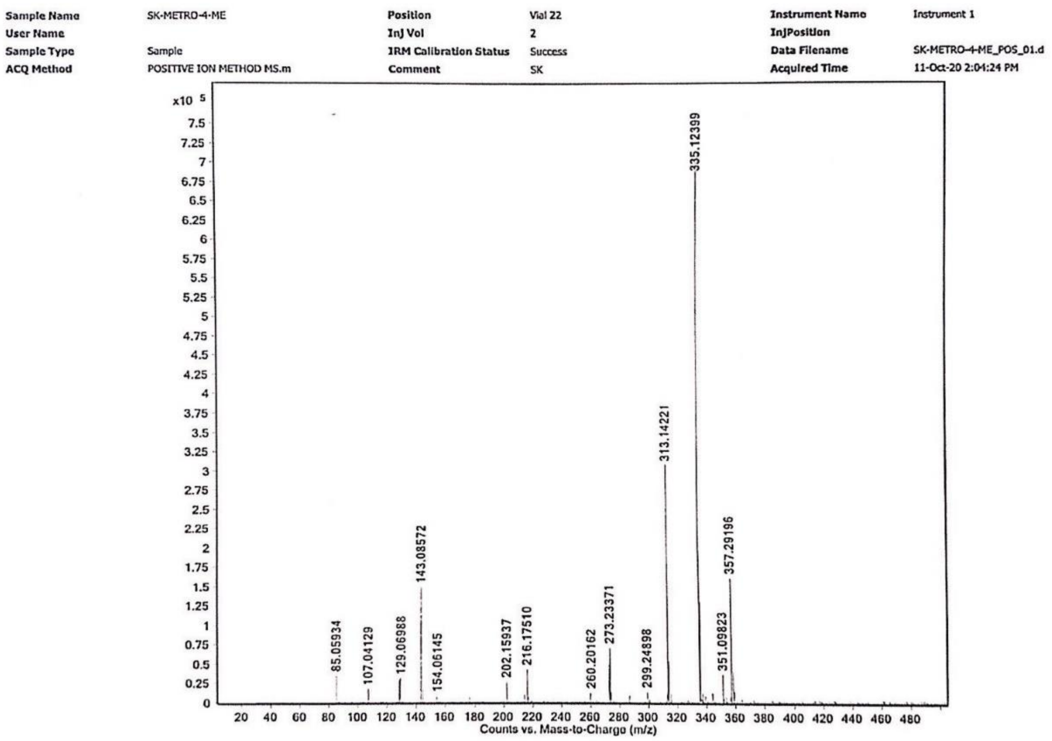

S29: HRMS spectrum of compound **5g**

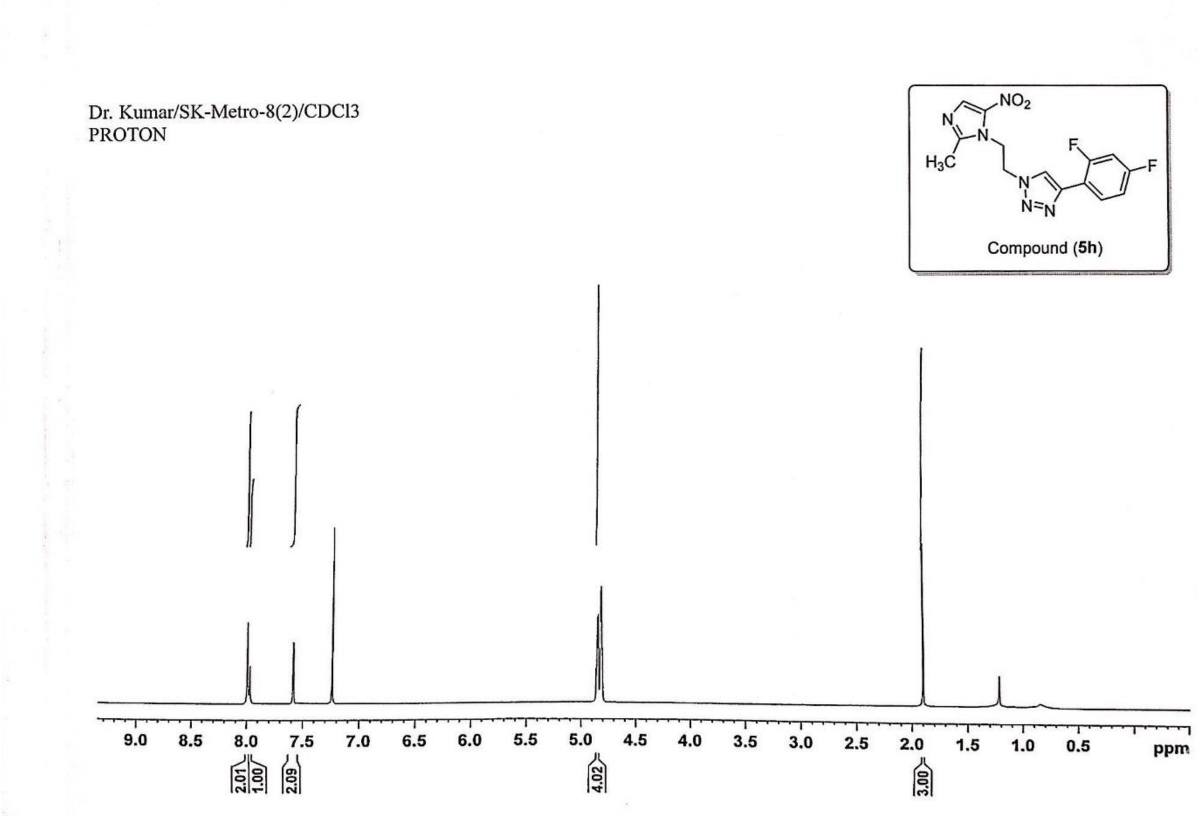

S30: ^{1}H-NMR spectrum (600 MHz, $CDCl_3$) of compound **5h**

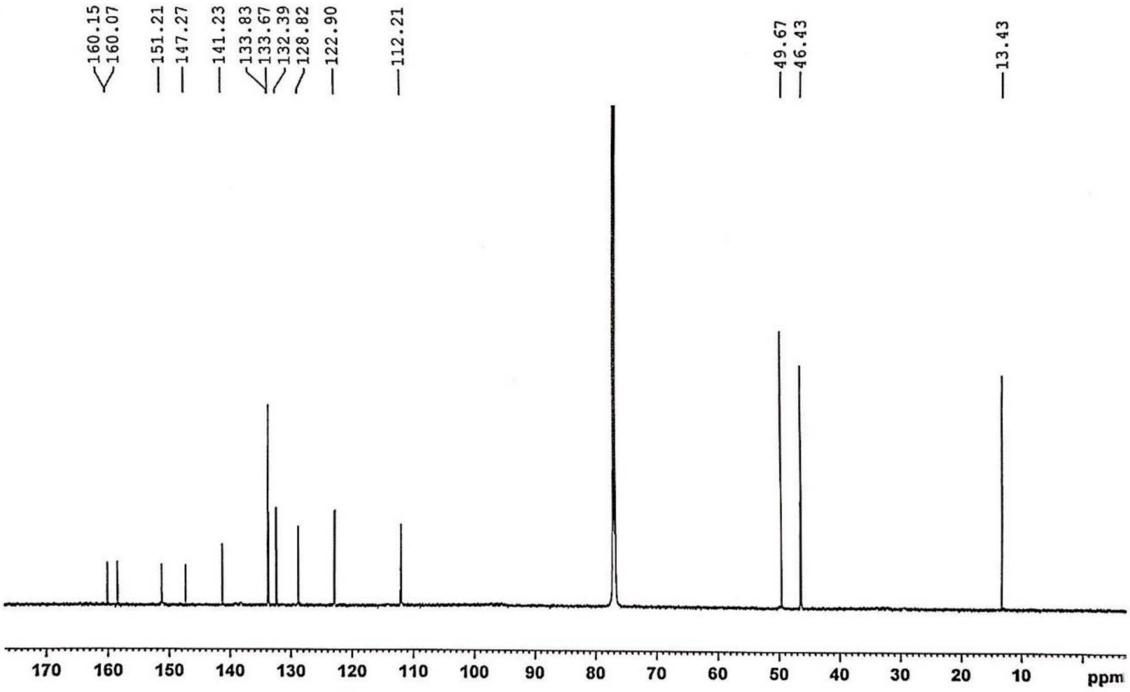

S31: ^{13}C-NMR spectrum (150 MHz, $CDCl_3$) of compound **5h**

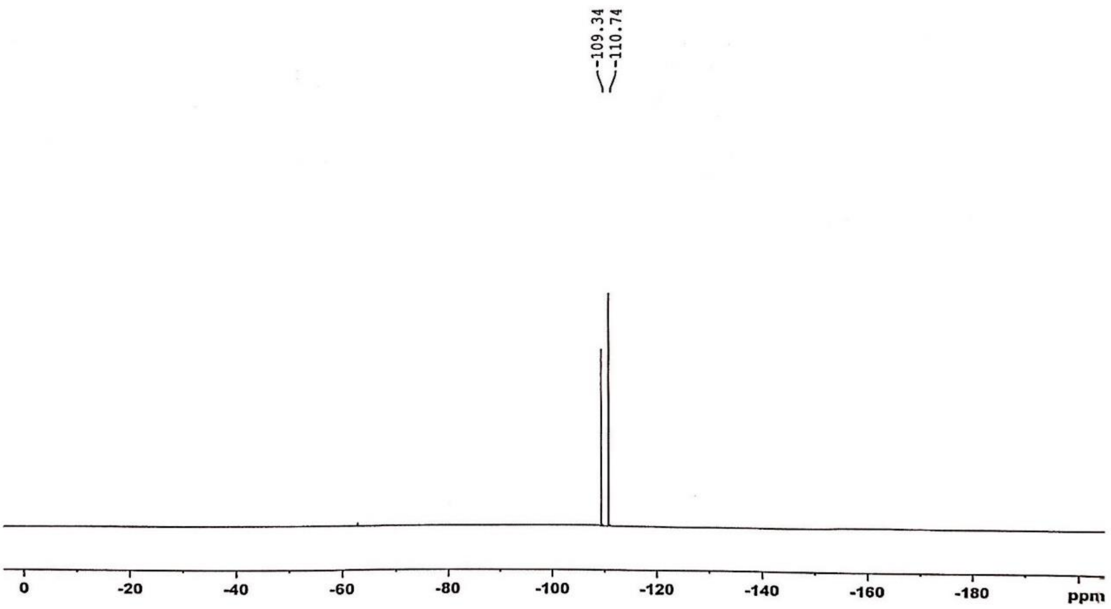

S32: ^{19}F-NMR spectrum (564 MHz, $CDCl_3$) of compound **5h**

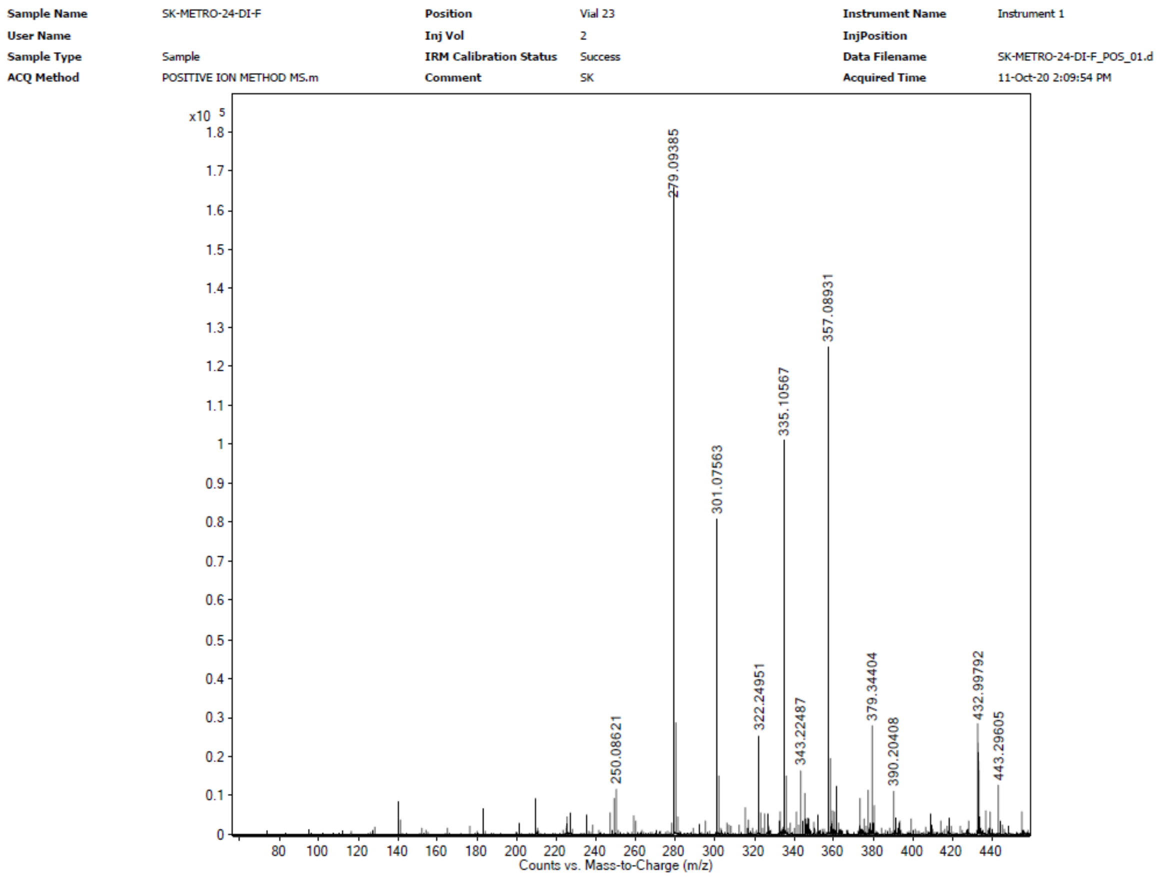

S33: HRMS spectrum of compound **5h**

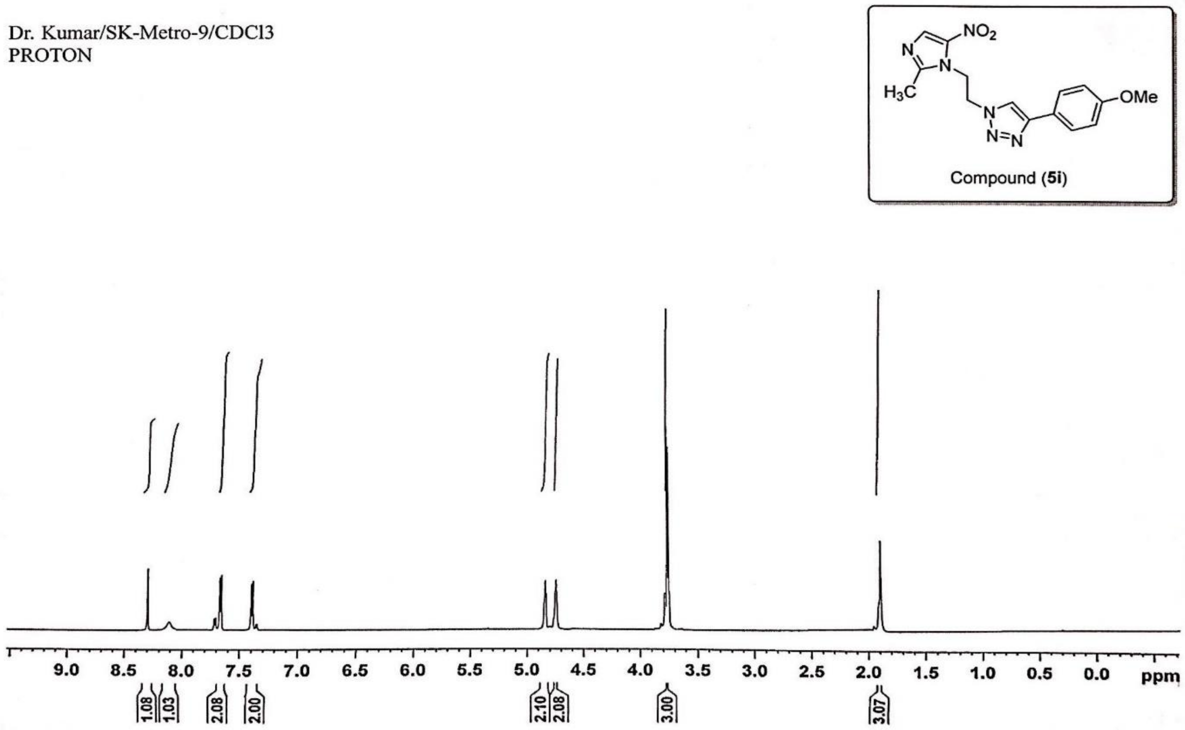

S34: ^{1}H-NMR spectrum (600 MHz, $CDCl_3$) of compound **5i**

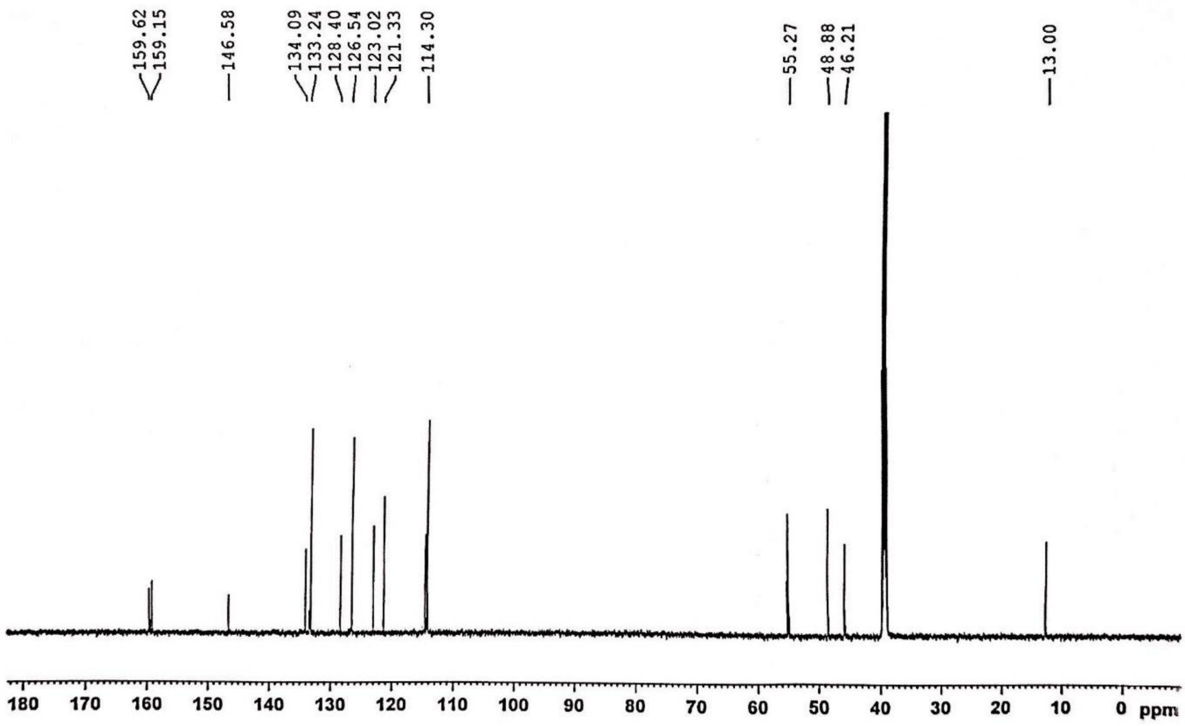

S35: ^{13}C-NMR spectrum (150 MHz, $CDCl_3$) of compound **5i**

**Sample Name**	SK-METRO-4-OME	**Position**	Vial 24	**Instrument Name**	Instrument 1
**User Name**		**Inj Vol**	2	**InjPosition**	
**Sample Type**	Sample	**IRM Calibration Status**	Success	**Data Filename**	SK-METRO-4-OME_POS_01.d
**ACQ Method**	POSITIVE ION METHOD MS.m	**Comment**	SK	**Acquired Time**	11-Oct-20 2:15:27 PM

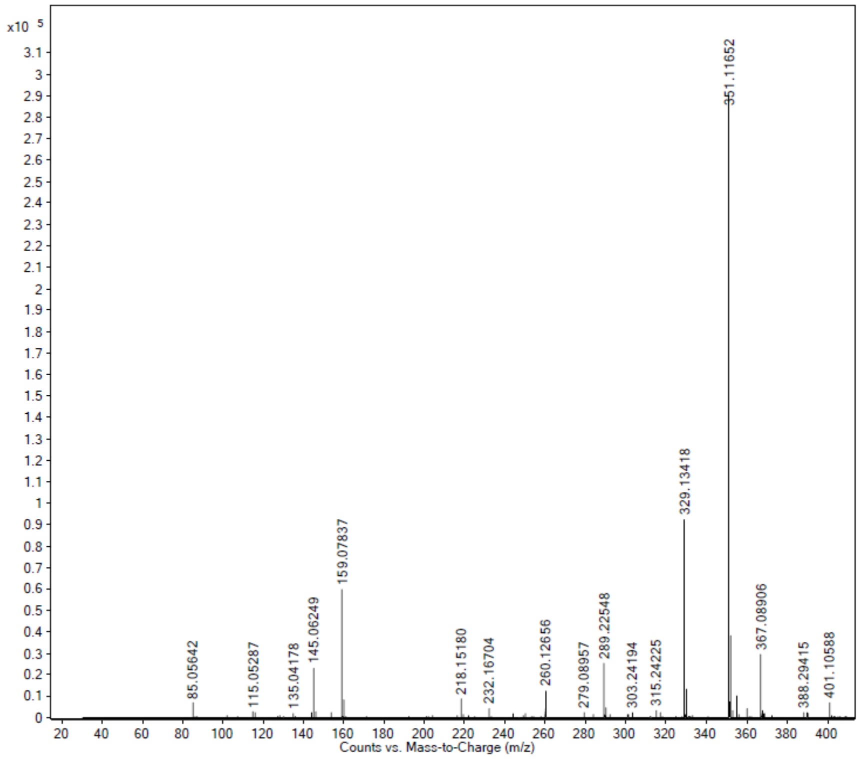

S36: HRMS spectrum of compound **5i**

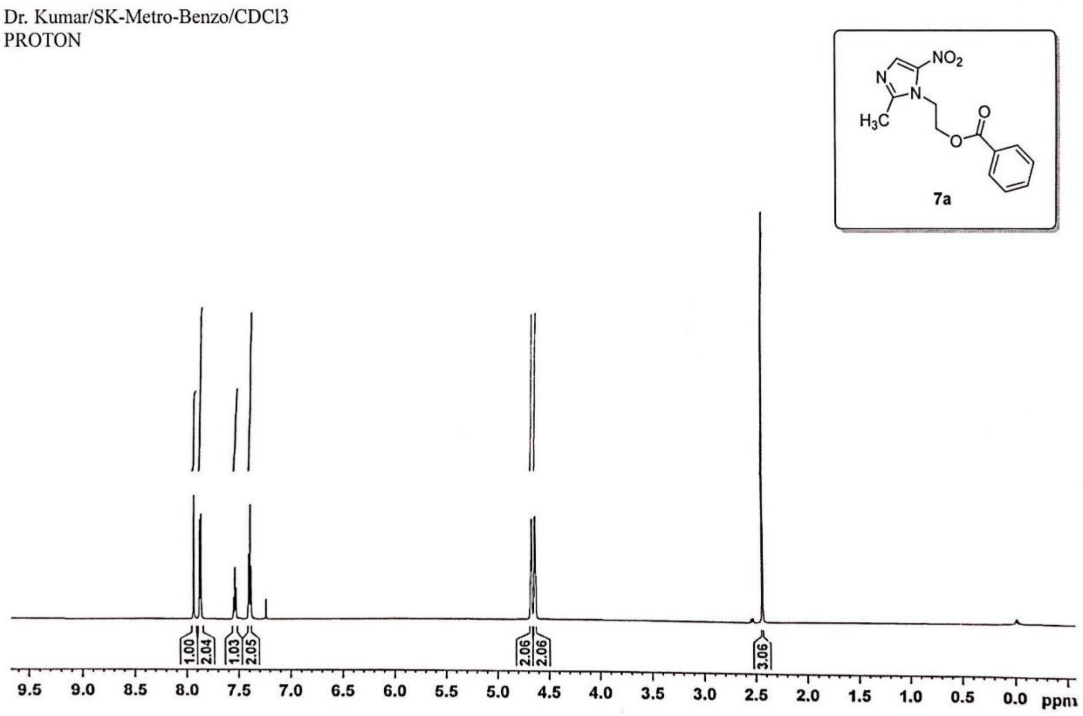

S37: ^{1}H-NMR spectrum (600 MHz, $CDCl_3$) of compound **7a**

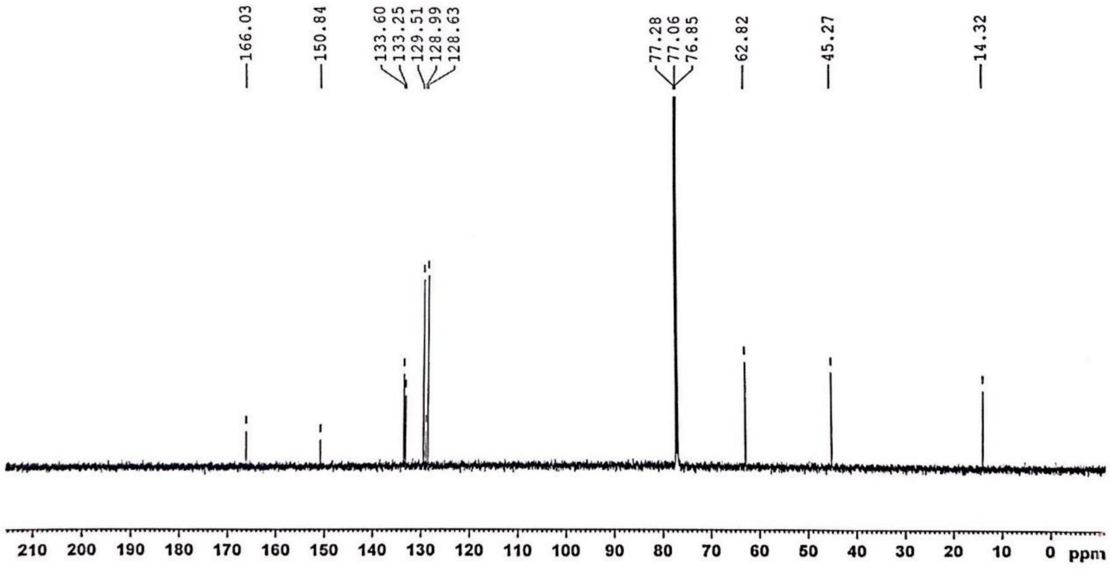

S38: ^{13}C-NMR spectrum (150 MHz, $CDCl_3$) of compound **7a**

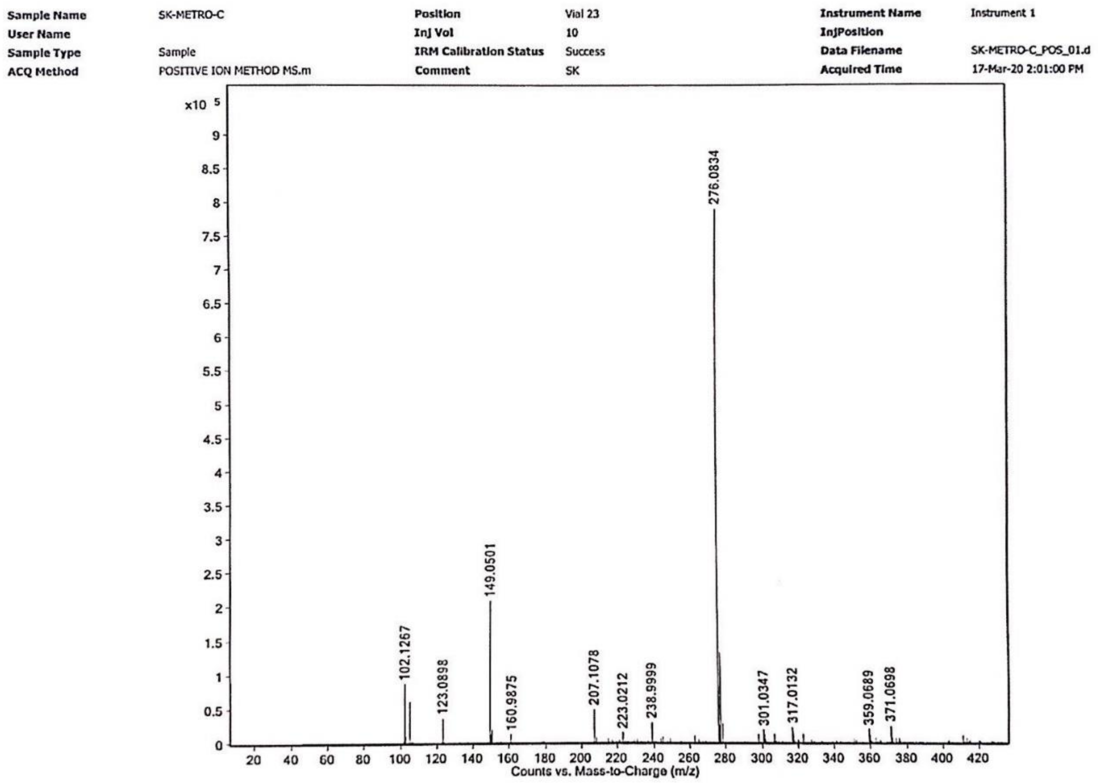

S39: HRMS spectrum of compound **7a**

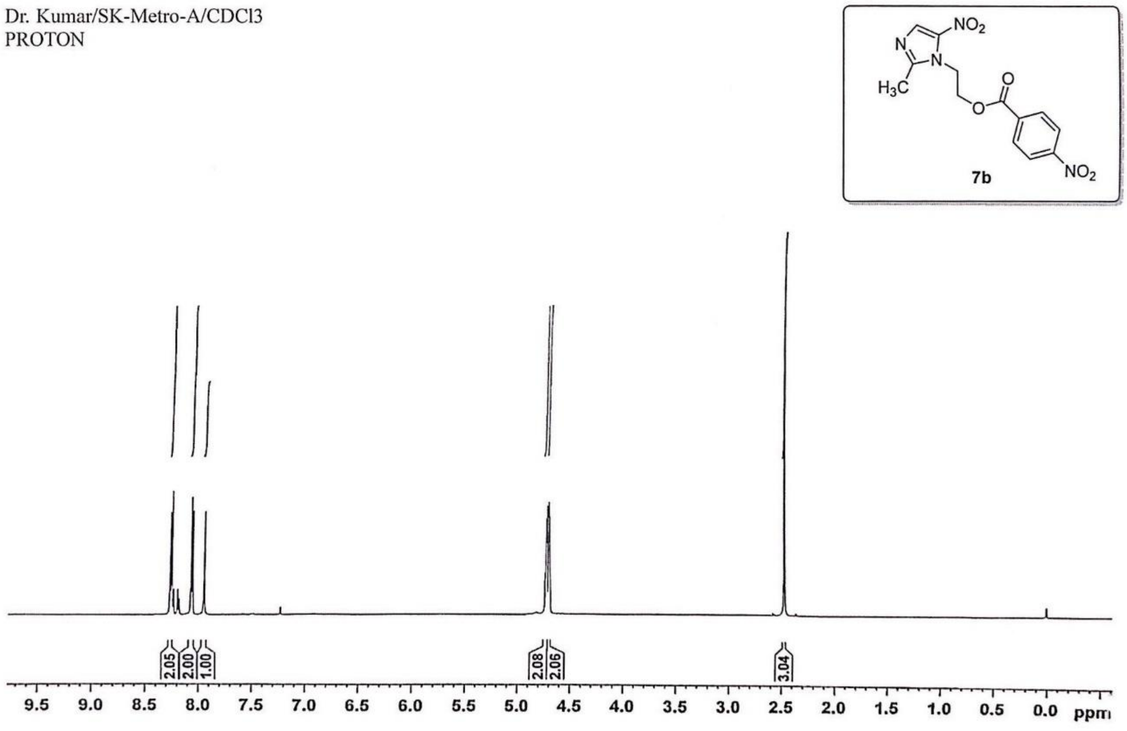

S40: ^{1}H-NMR spectrum (600 MHz, $CDCl_3$) of compound **7b**

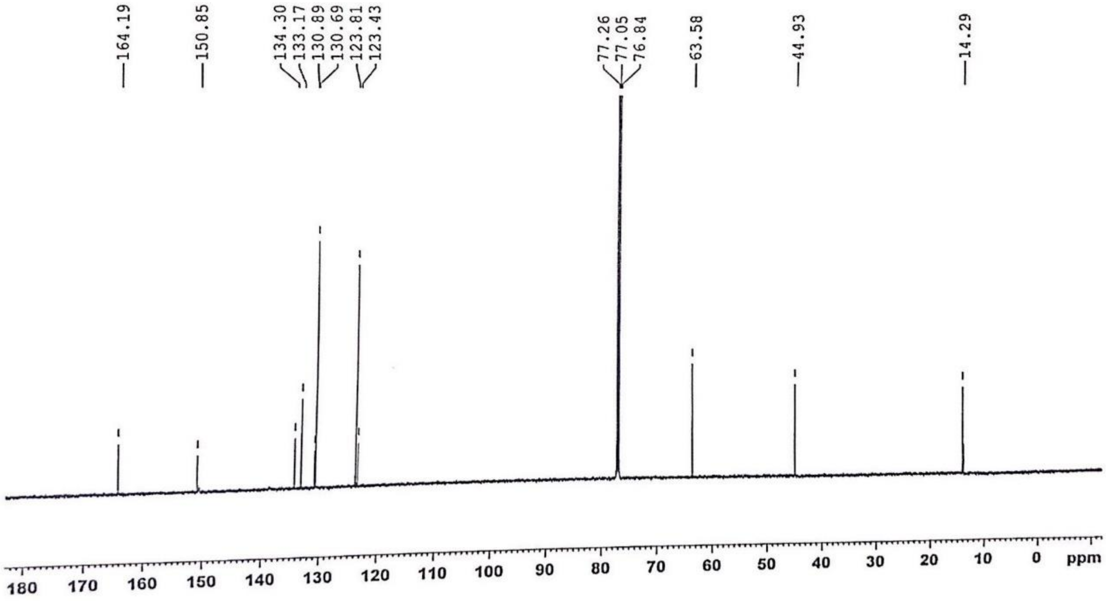

S41: ^{13}C-NMR spectrum (150 MHz, $CDCl_3$) of compound **7b**

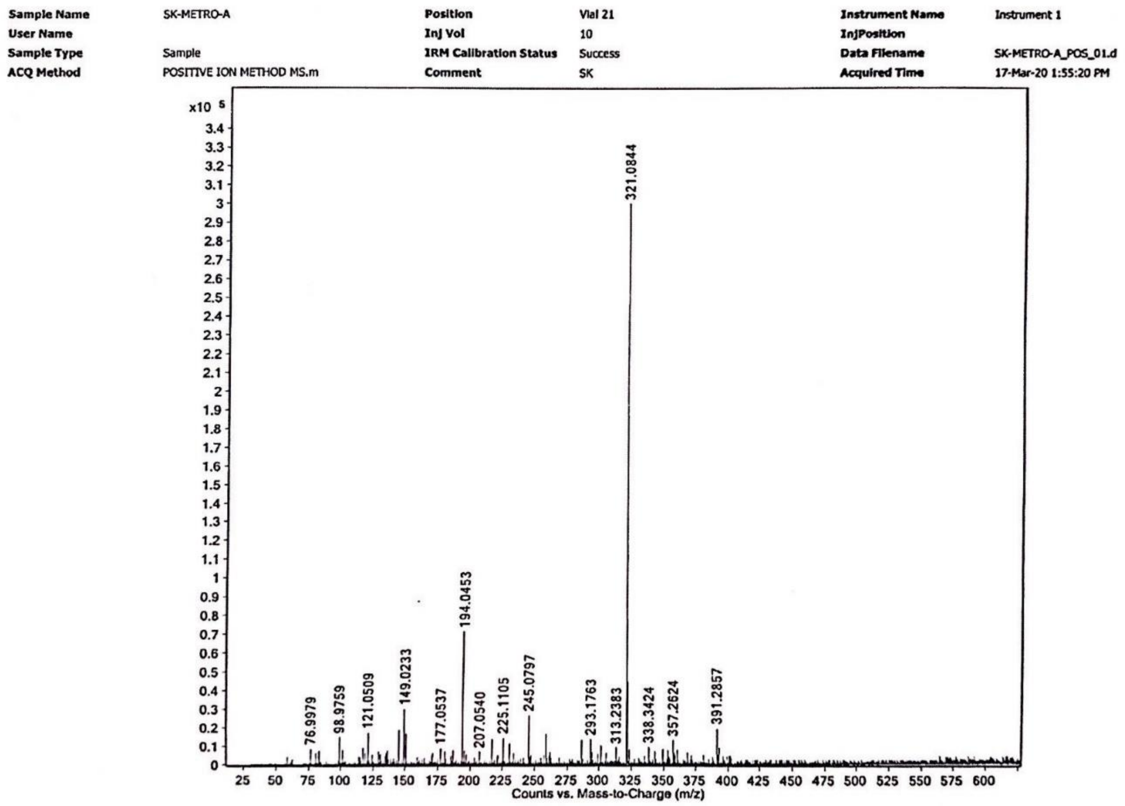

S42: HRMS spectrum of compound **7b**

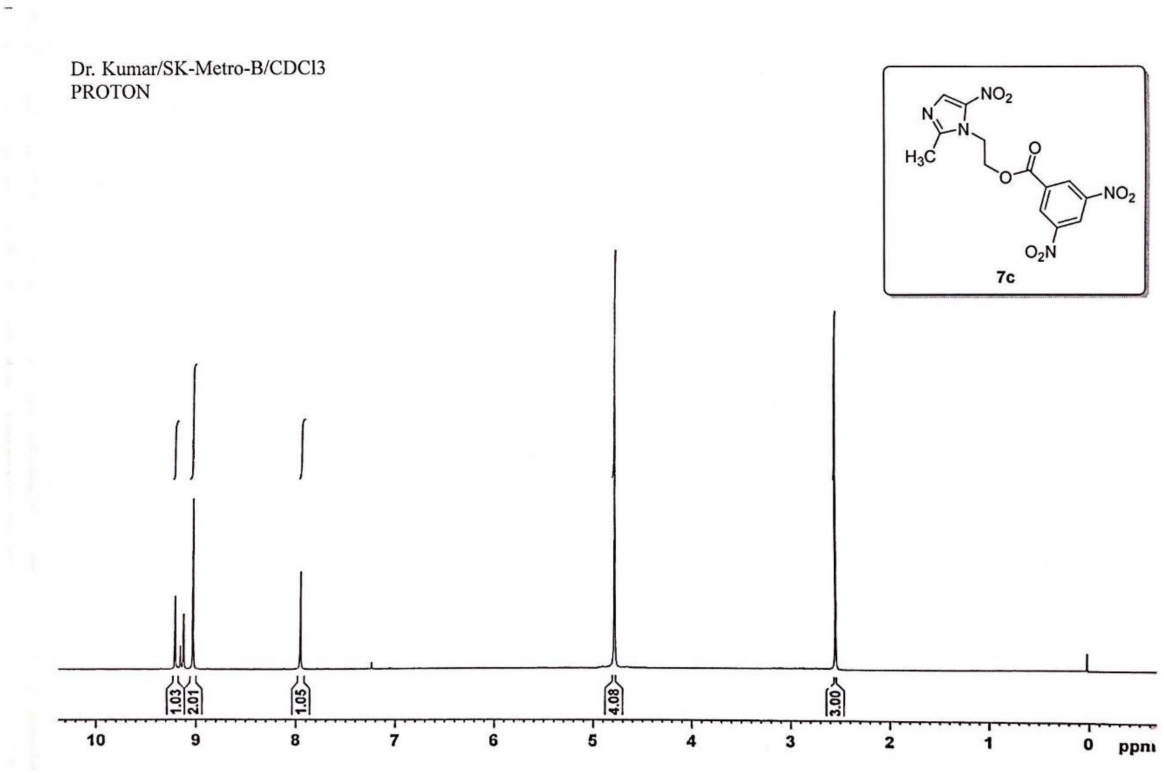

S43: ^{1}H-NMR spectrum (600 MHz, $CDCl_3$) of compound **7c**

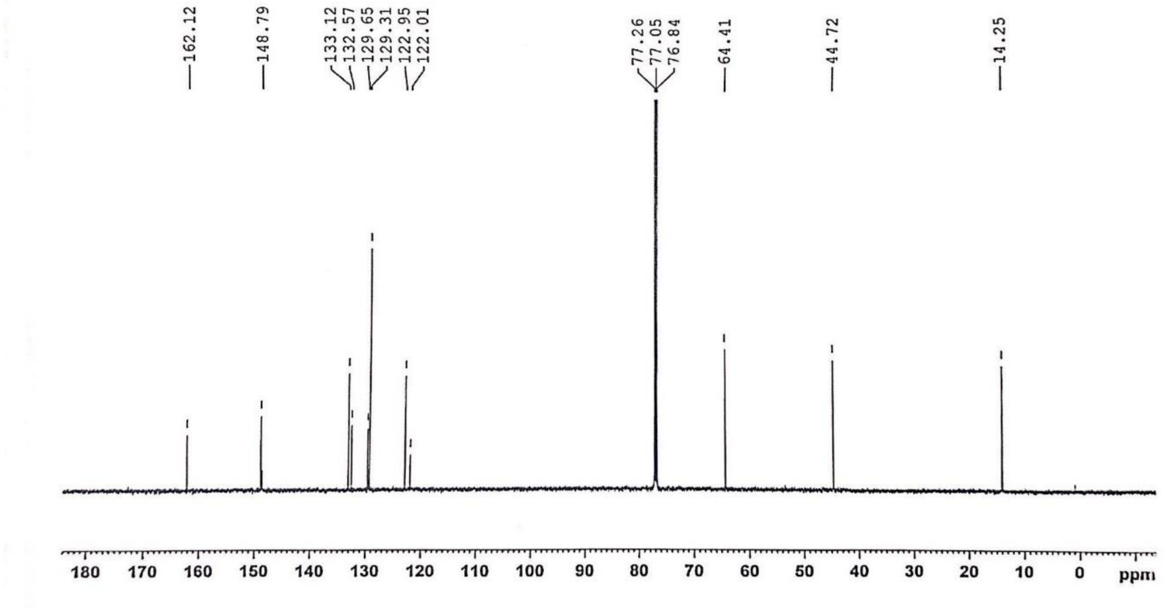

S44: ^{13}C-NMR spectrum (150 MHz, $CDCl_3$) of compound **7c**

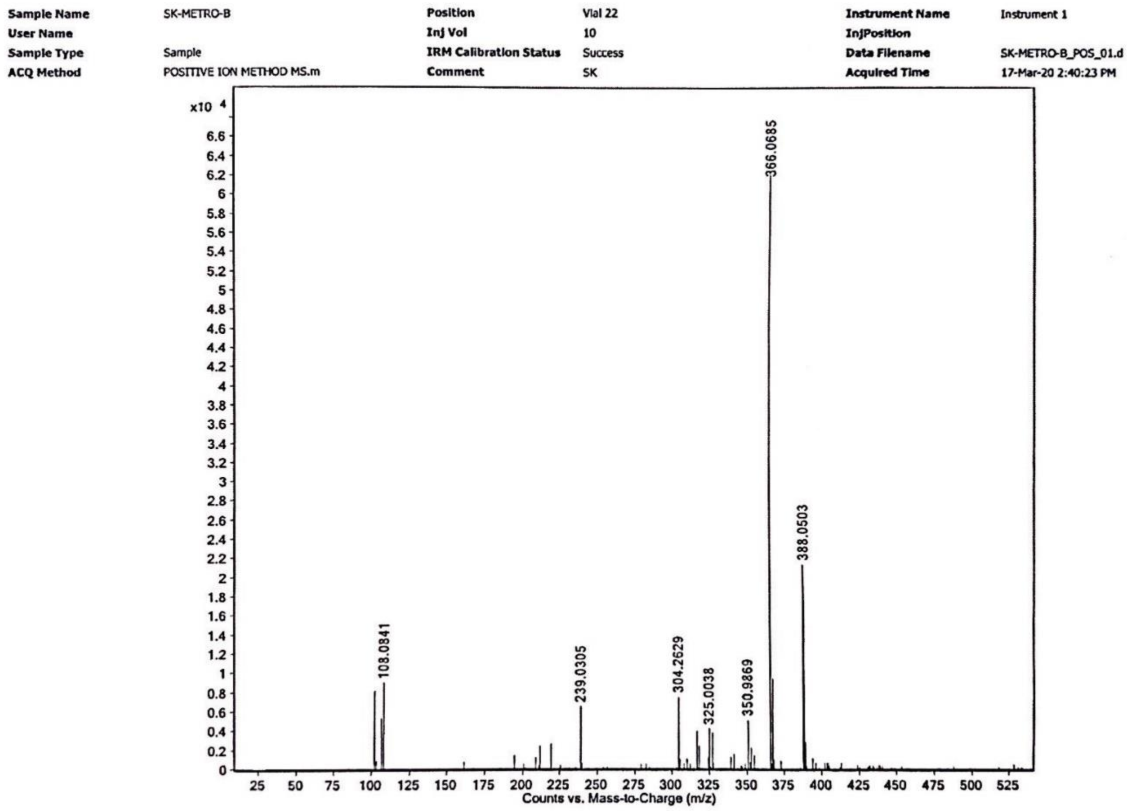

S45: HRMS spectrum of compound **7c**

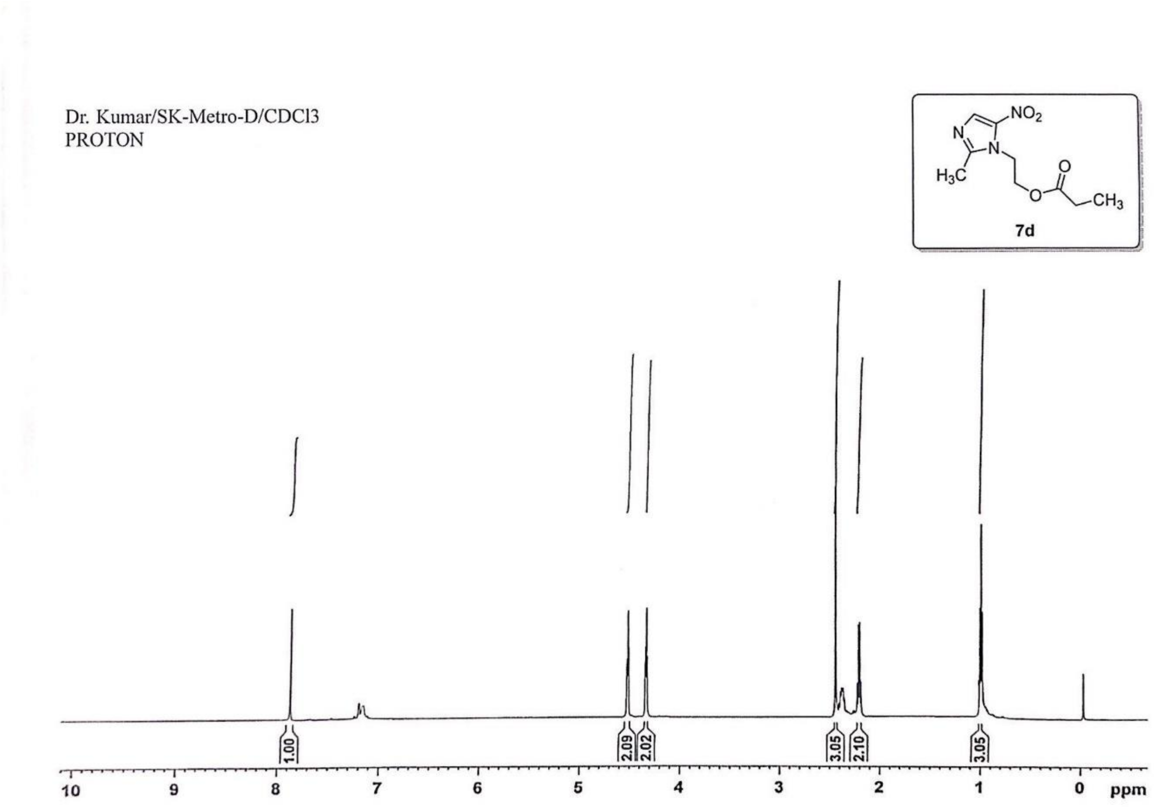

S46: ^{1}H-NMR spectrum (600 MHz, $CDCl_3$) of compound **7d**

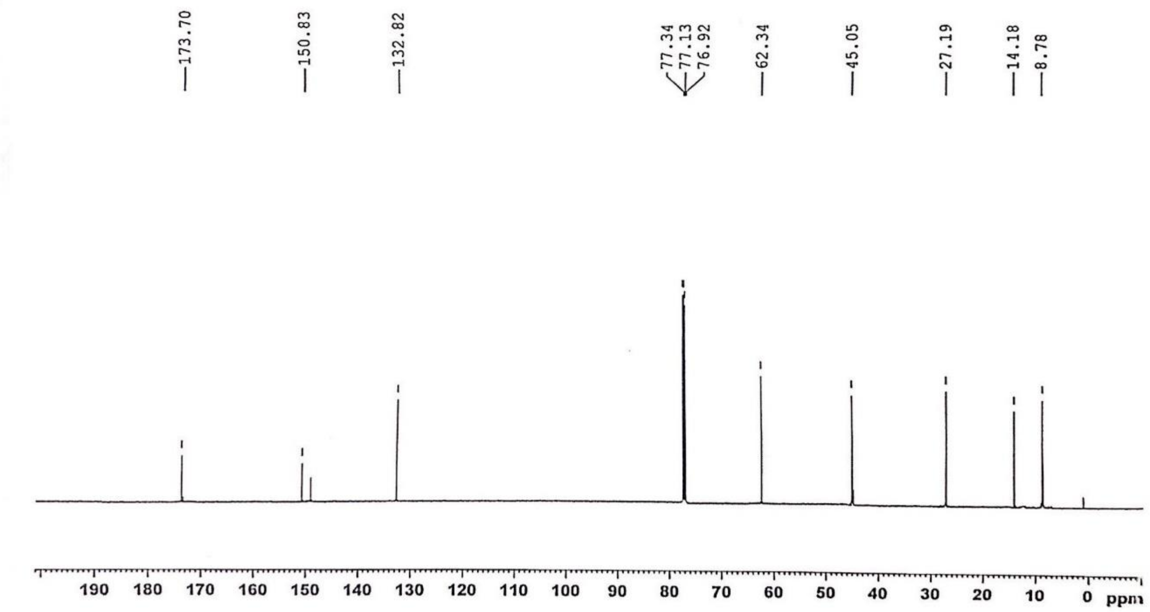

S47: ^{13}C-NMR spectrum (150 MHz, $CDCl_3$) of compound **7d**

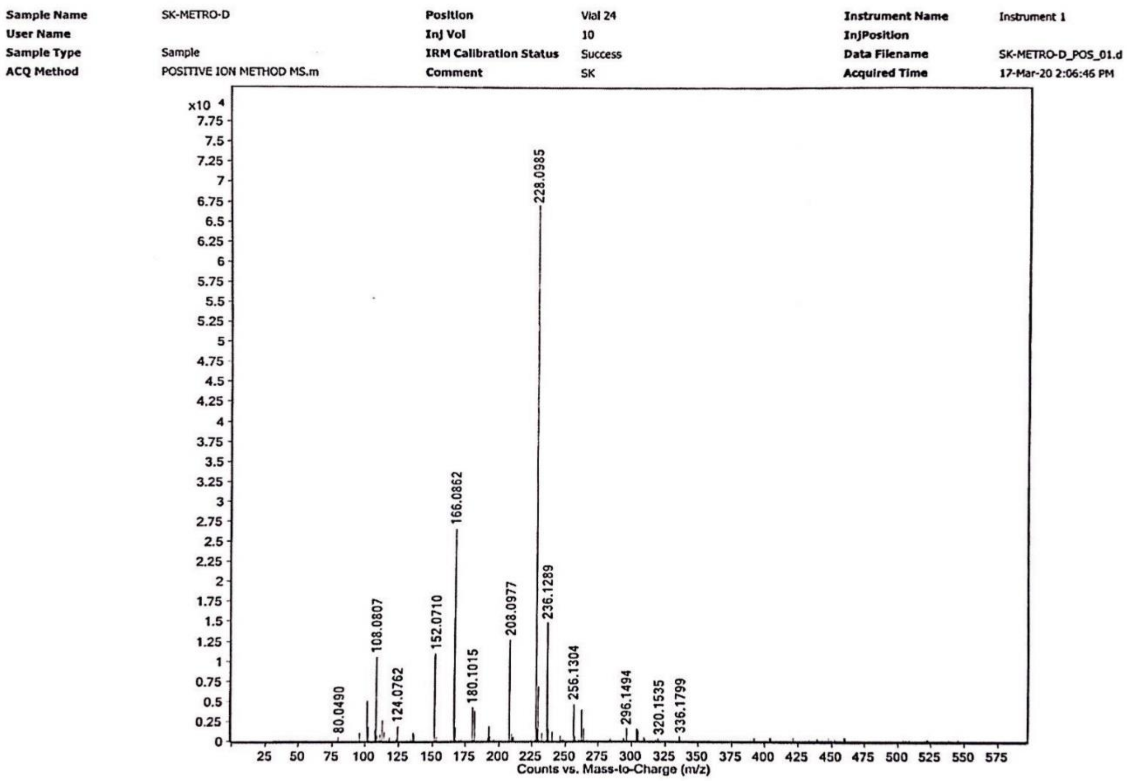

S48: HRMS spectrum of compound **7d**

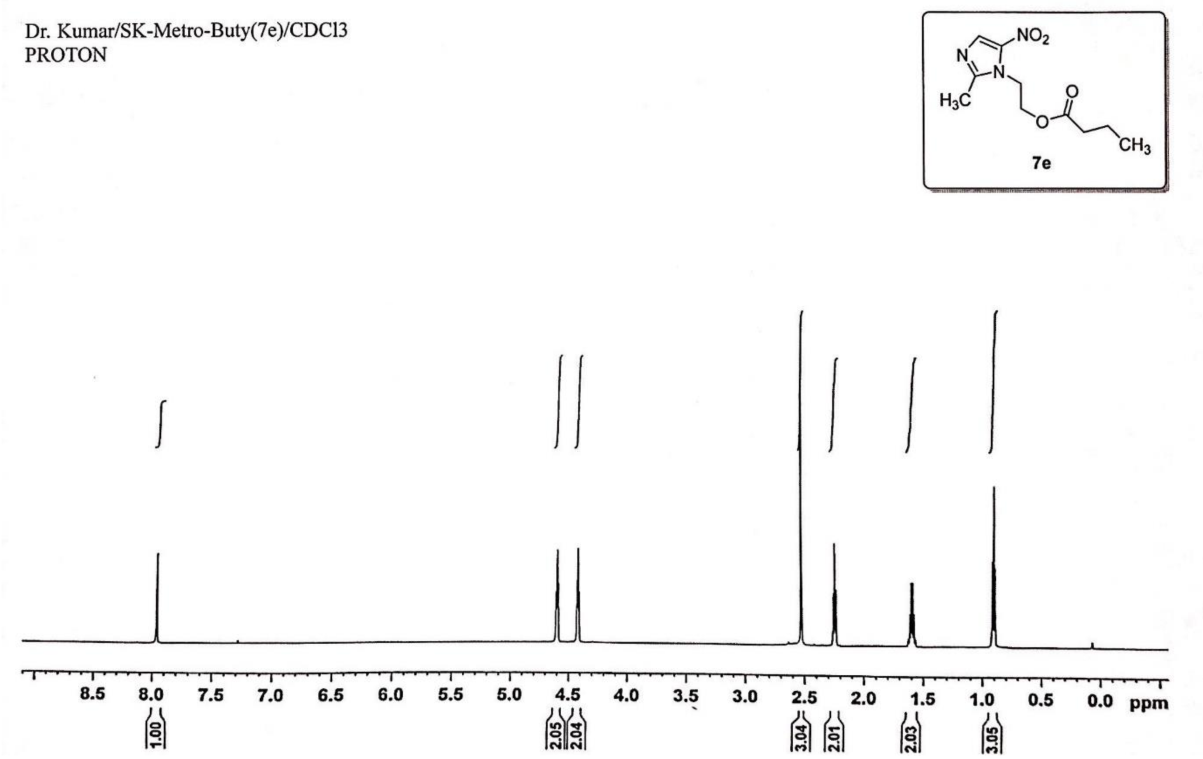

S49: ^{1}H-NMR spectrum (600 MHz, $CDCl_3$) of compound **7e**

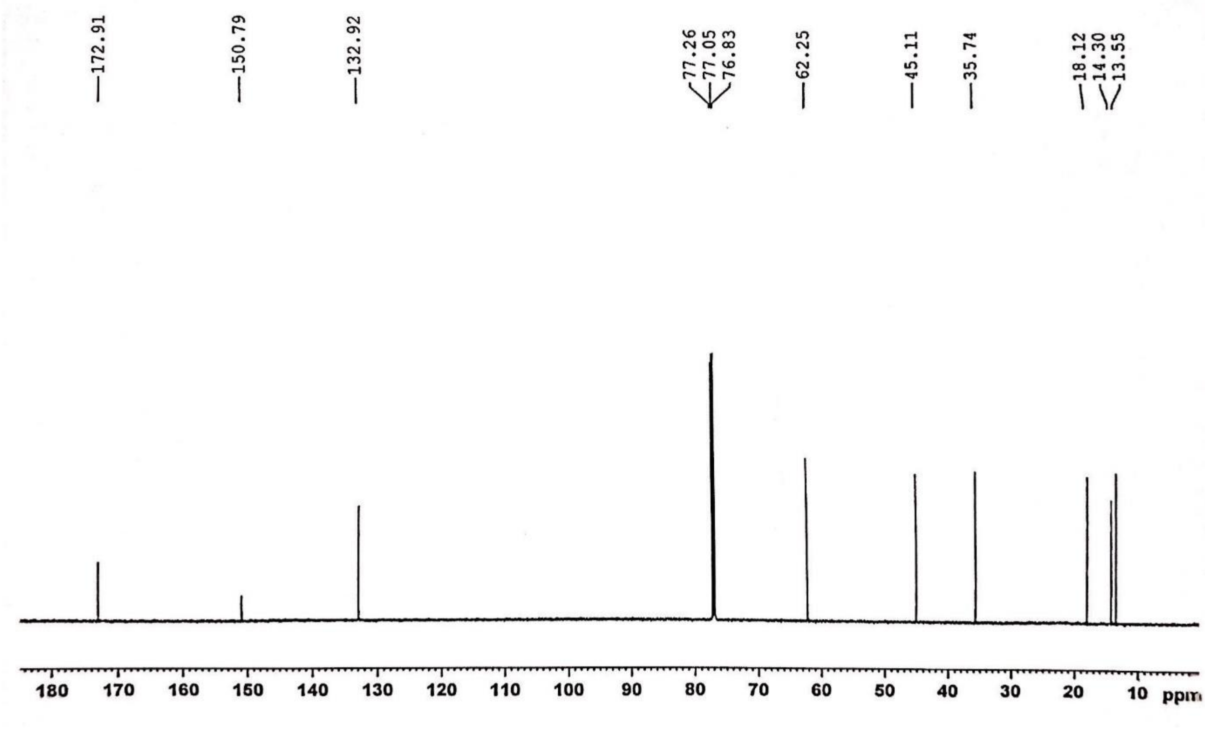

S50: ^{13}C-NMR spectrum (150 MHz, $CDCl_3$) of compound **7e**

**Sample Name**	Unavailable	**Position**	Unavailable	**Instrument Name**	Unavailable
**User Name**	Unavailable	**Inj Vol**	Unavailable	**InjPosition**	Unavailable
**Sample Type**	Unavailable	**IRM Calibration Status**	Success	**Data Filename**	SK-METRO-7e_001.d
**ACQ Method**		**Comment**	Sample information is unavailable	**Acquired Time**	Unavailable

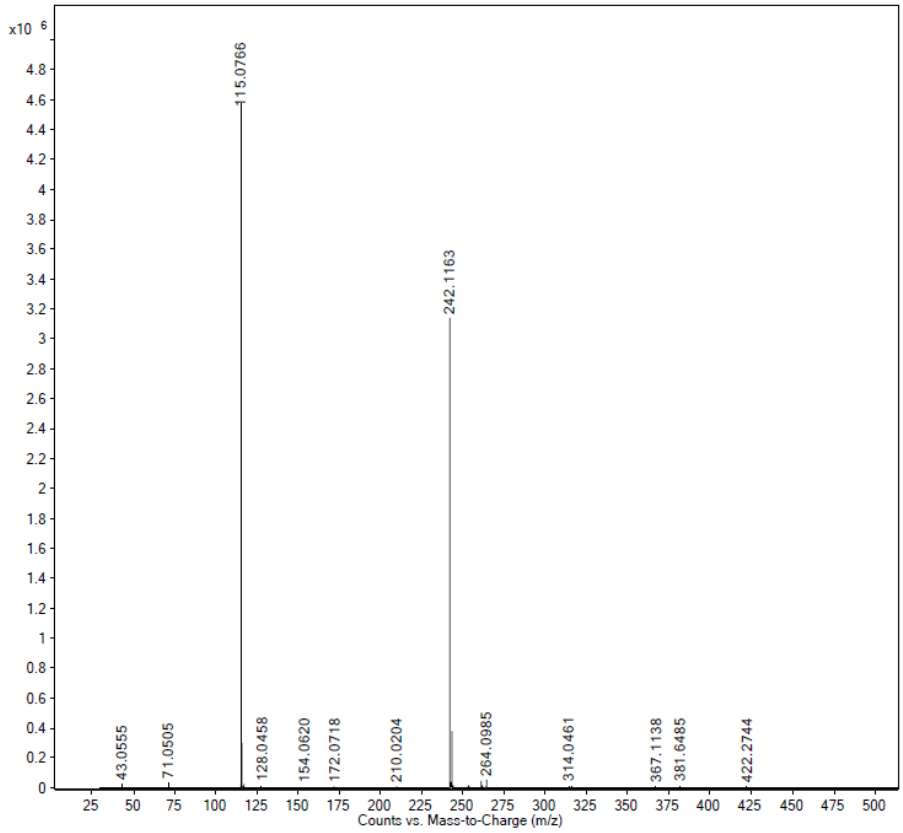

S51: HRMS spectrum of compound **7e**
